# Laser microdissection transcriptome data derived gene regulatory networks of developing rice endosperm revealed tissue- and stage-specific regulators modulating starch metabolism

**DOI:** 10.1007/s11103-021-01225-w

**Published:** 2022-01-31

**Authors:** Tsutomu Ishimaru, Sabiha Parween, Yuhi Saito, Takehiro Masumura, Motohiko Kondo, Nese Sreenivasulu

**Affiliations:** 1 NARO Institute of Crop Science, NARO, 2-1-18 Kannondai, Tsukuba, Ibaraki 305-8518 Japan; 2 Hokuriku Research Station, Central Region Agricultural Research Center, National Agriculture and Food Research Organization (CARC/NARO), 1-2-1 Inada, Joetsu, Niigata 941-0193 Japan; 3 International Rice Research Institute (IRRI), DAPO Box 7777, Metro Manila, The Philippines; 4 Graduate School of Life and Environmental Science, Kyoto Prefectural University, Shimogamo, Sakyo-ku, Kyoto, 606-8522 Japan; 5 Graduate School of Bioagricultural Sciences, Nagoya University, Furo, Chikusa, Nagoya, 464-8601 Japan

**Keywords:** Aleurone cells, Developing endosperm, Laser microdissection, Rice, Starchy endosperm, Storage compounds, Transcriptome

## Abstract

**Key message:**

Laser microdissection applied on the developing rice endosperm revealed tissue- and stage-specific regulators modulating programmed cell death and desiccation tolerance mechanisms in the central starchy endosperm following starch metabolism.

**Abstract:**

Rice (*Oryza sativa* L.) filial seed tissues are heterozygous in its function, which accumulate distinct storage compounds spatially in starchy endosperm and aleurone. In this study, we identified the 18 tissue- and stage-specific gene co-regulons in the developing endosperm by isolating four fine tissues dorsal aleurone layer (AL), central starchy endosperm (CSE), dorsal starchy endosperm (DSE), and lateral starchy endosperm (LSE) at two developmental stages (7 days after flowering, DAF and 12DAF) using laser microdissection (LM) coupled with gene expression analysis of a 44 K microarray. The derived co-expression regulatory networks depict that distinct set of starch biosynthesis genes expressed preferentially at first in CSE at 7 DAF and extend its spatial expression to LSE and DSE by 12 DAF. Interestingly, along with the peak of starch metabolism we noticed accumulation of transcripts related to phospholipid and glycolipid metabolism in CSE during 12 DAF. The spatial distribution of starch accumulation in distinct zones of starchy endosperm contains specific transcriptional factors and hormonal-regulated genes. Genes related to programmed cell death (PCD) were specifically expressed in CSE at 12DAF, when starch accumulation was already completed in that tissue. The aleurone layer present in the outermost endosperm accumulates transcripts of lipid, tricarboxylic acid metabolism, several transporters, while starch metabolism and PCD is not pronounced. These regulatory cascades are likely to play a critical role in determining the positional fate of cells and offer novel insights into the molecular physiological mechanisms of endosperm development from early to middle storage phase.

**Supplementary Information:**

The online version contains supplementary material available at 10.1007/s11103-021-01225-w.

## Introduction

Rice (*Oryza sativa* L.) is a staple food for providing calories to more than half of the world’s population (Carriger and Vallee 2007). Rice grain is rich with major storage compounds such as starch and proteins with minor representation of lipid, minerals and phytonutrients. Many vital nutritional storage compounds do accumulate in aleurone layer (AL), which is removed upon milling during the processing of the mature grain. As a result, the milled starchy endosperm (SE) is predominantly rich in starch (up to 90%) and different storage proteins (6–8%) primarily used for human consumption to provide the necessary calories (Itani et al. 2002; Tanaka et al. 1995). It is important to understand the underlying molecular-physiological process occurring during AL and SE development, critical to improve rice grain quality. 

Rice endosperm consists of different zones conferring distinct physiological processes during the onset of development. Aleurone cells are the first filial tissue that receives the unloaded nutrients at the outermost filial endosperm tissue (Oparka and Gates 1981), which will be rerouted to endosperm. Aleurone cells start to differentiate at 5 DAF (Ishimaru et al. 2003) concomitantly with the start of vital nutritional storage compound accumulation such as lipids, accumulation of globulins, micronutrients, vitamins, antioxidants and dietary fibre (Tanaka et al. 1995). Aleurone cells remain viable throughout seed development to ensure the supply of sucrose and other nutrients through active transporters. 5–7 DAF corresponds to the initial stage of starch accumulation (Hoshikawa 1968; Ishimaru et al. 2003), with the metabolic switch of sucrose cleavage from invertase to sucrose synthase (Hirose et al. 2002, 2008) and channeling the hexoses to activate starch metabolism (Hirose and Terao 2004). Accumulation of starch and storage proteins proceeds asynchronously among SE zones. During 5–8 DAF, starch accumulation predominantly occurs at the central zone of starchy endosperm (CSE), and then starch accumulation spreads to the outer zone such as lateral and dorsal starchy endosperm (LSE and DSE, respectively) (Hoshikawa 1968; Ishimaru et al. 2003). Amylose content remains highest in the CSE zone among endosperm tissues (Itani et al. 2002). Spatial and temporal accumulation of starch is concomitantly associated with changes in water distribution in the developing endosperm (Horigane et al. 2001; Ishimaru et al. 2009). Programmed cell death (PCD) of endosperm initiates in CSE zone, then proceeds to peripheral zones (Kobayashi et al. 2013), suggesting that PCD of SE is coordinated with the completion of the starch granule packing (Xu et al. 2010). On the other hand, storage proteins are preferentially accumulated in the peripheral layer of starchy endosperm such as LSE zone, while they are not accumulated so much in DSE zone (Saito et al. 2008). Spatial and temporal preference in accumulation of storage compounds differs even among zones of SE.

Because of the spatial differences in its function and diversified storage accumulation patterns among zones of developing endosperm, it is important to dissect the heterogeneous endosperm tissue into compartments to derive tissue- and stage-specific gene regulatory networks likely to shed interesting insights into molecular-physiological process occur during AL and SE development. LM is a powerful tool for dissecting targeted specific cells from heterogeneous tissue viewed under a microscope, using an intense laser beam (Emmert-Buck et al. 1996). With LM, we previously succeeded in developing a method for obtaining high-quality RNA from developing rice endosperm, facilitating precise expression analysis (Ishimaru et al. 2007). Note that separating these specific zones of endosperm cells such as AL, CSE, DSE, and LSE is impossible through manual dissection. Our previous LM study coupled with qRT-PCR on developing endosperm at 7DAF revealed that the distinct metabolic pathways exist between AL and SE zones; AL predominantly expressed mRNAs for the sucrose uptake, sucrose cleavage, TCA cycle, and oxidative phosphorylation supported by large numbers of mitochondria and presence of oxygen (Ishimaru et al. 2015). On the contrary, the expression levels of ADP-glucose pyrophosphorylase (*AGPL2* and *AGPS2b*) in each SE tissue spatially corresponded to the distribution of starch granules within the endosperm tissues (Ishimaru et al. 2015). This preliminary LM study infers the dynamic and coordinated changes in expression of genes in relation to distinct storage compounds accumulated in the zones of developing rice endosperm. Indeed, previous transcriptome and proteome studies using entire starchy endosperm suggest important roles of central carbon and starch metabolism, protein and amino acid metabolism, carbon fixation, alcoholic fermentation, hormone regulation, and transcription factors temporally regulated during rice endosperm development (Gao et al. 2013; Xu et al. 2010; Xue et al. 2012). Wu et al. (2020) applied LM technology to the fine maternal tissues and developing endosperm in rice to identify the important transcriptional factors that control endosperm development, but we do not understand spatial expression patterns regulating distinct storage compounds among fine endosperm zones. Mutant studies with altered rice endosperm development have revealed critical regulators related to aleurone cell specification, starch synthesis, storage protein accumulation (See review, Zhou et al. 2013).

The present study aims to reveal tissue-specific and stage-preference gene regulatory networks among different zones of developing SE tissue fractions as well in AL tissue fractions, which exhibit different functions and accumulate distinct storage compounds. LM was used to isolate the specific endosperm zones from developing grains of 7 DAF and 12 DAF as representative of early and middle storage phase, respectively. Transcriptome derived molecular physiological mechanisms of endosperm development from early to middle storage phase is unraveled and distinct regulators influencing distinct storage compounds and PCD regulation is discussed. 

## Material and methods

### Plant materials

A Japonica Group rice cultivar, ‘Nipponbare’ (*Oryza sativa* L.), was germinated and a four-week seedling was transplanted to 0.02 m^2^ pots with a fertilizer application of 0.5–2.3–2.2 g of N–P_2_O_5_–K_2_O as basal dressing. Plants were grown outdoors, then pots were transferred into a naturally illuminated temperature-controlled chamber at the booting stage. Day (13 h) and night (11 h) air temperatures were maintained at 26 °C and 20 °C, respectively until maturity. Top dressing (0.4 g of nitrogen per pot) and chemical spraying were applied as necessary. Flowering spikelets located from the first to the fourth primary rachis branches counted from the top of the panicle, which are represented as ‘superior caryopses’ (Ishimaru et al. 2003) were marked with fine-tipped pens. Plants were kept disease-free with the application of appropriate chemicals.

### Stereo microscope

Entire developing grain from 0 to 20 days after flowering (DAF) and matured kernel were viewed under a stereo microscope (SZX10, Olympus) and photographed with a digital camera (E-330, Olympus). Median transverse sections (1.0–1.5 mm thickness) of developing caryopses at 7 and 12DAF, and maturity were made by a sharp razor blade and photographed.

### Transmission electron microscope (TEM)

Developing endosperm zones of AL, CSE, DSE, and LSE at seven days and 12 days after flowering (DAF) was observed following the method of Ishimaru et al. (2019).

### Laser microdissection (LM)

Fixation, embedding, and dissection of fine endosperm tissues by LM were based on the method of Ishimaru et al. (2007). Developing caryopsis at 7 and 12DAF were prepared for LM with three biological replicates. Aleurone layer at the dorsal side (AL), center, dorsal, and lateral starchy endosperm (CSE, DSE, and LSE, respectively) were microdissected with an AS LMD system (Leica Microsystems, Wetzlar, Germany) as described by Ishimaru et al. (2019).

### One-color microarray analysis

Extraction of ribonucleic acid (RNA), integrity assessment, and quantification of RNA were conducted following the methods of Ishimaru et al. (2007). Microarray experiment was conducted based on the method of Takehisa et al. (2012). Total RNA (2.5 ng) was amplified to obtain complementary RNA (cRNA) using a Quick Amp Labeling kit and labelled using One-color (Agilent technologies) cyanine-3 (Cy3)-CTP, according to the modified manufacturer’s instruction. The Cy3-labeled cRNA was purified by Rneasy Mini Kit (Qiagen). Concentration of cRNA was quantified by a NanoDrop ND-1000 UV–VIS spectrophotometer (NanoDrop Technologies). A total of 900 ng Cy3-labeled cRNAs were fragmented and hybridized on a slide glass of rice 4 × 44 K microarray RAP-DB (G2519F#15241; Agilent Technologies). Hybridization and washing of the hybridized slides were performed according to the manufacturer’s instructions. Slides were scanned on an Agilent G2505B DNA microarray scanner, and background of the Cy3 raw signals was corrected using the Feature Extraction (ver. 10.5.1.1, Agilent Technologies).

### Calculation of differential expressed genes

The single channels Agilent microarray expression data of 12 microdissected samples of AL, DSE, CSE and LSE at 7DAF and 12 DAF of three replicates were used for differential expression analysis (DEGs). The differentially expressed genes (DEGs) were calculated in three sets (Supplementary Table S1) using the R package limma (Ritchie et al. 2015; Smyth 2004) which uses the linear and empirical based methods. At first DEGs calculated within 7DAF between different tissue types such as, AL, DSE, CSE, and LSE, subsequently between different tissue types within 12 DAF. Secondly, across all tissue types of 7DAF vs 12DAF and thirdly 7DAF vs 12DAF tissues individually. All the sets were combined and only unique probes of ~ 15 k were retained with log_2_ fold change |> + -1| based on the P-value and adjusted P-value (Benjamin and Hochberg) |< 0.05| (Supplementary Table S1).

## Co expression network construction

The DEGs extracted from a total of 24 samples, 3 replicates of 7DAF and 12DAF across AL, DSE, CSE and LSE tissues with log_2_ fold change |> + − 1| and P-value and adjusted P-value (Benjamin and Hochberg) |<= 0.05| resulted in identifying 15 k gene set (Supplementary Table S1). The normalized expression value of 15 k ~ unique set (Supplementary Table S2) was used as input for co-expression analysis. The R package WGCNA (Weighted Gene Coexpression Network Analysis) was used to calculate the co-expression modules following the signed network algorithm defined by Langfelder and Horvath (2008). To obtain tissue specific genes, complete linkage hierarchical clustering on each master module was done and the genes showing mixed expression values across the tissues were removed and heat map was constructed as rank based. The hub gene of each selected module was calculated using the softconnectivity function of WGCNA (Langfelder and Horvath 2008) algorithm (Supplementary Table S3). The visualization of the co-expression network was created by using Cystoscope software (Shannon et al. 2003).

## Enrichment analysis

The Enrichment analysis was done based on DEGs obtained from Supplementary Table S1. The input file for MapMan is significant log fold change of DEGs listed in Supplementary Table S1. For each of the selected modules significant enrichment analysis was done based on MapMan bins (Thimm et al. 2004) and significant numbers were sorted based on bonferroni corrected P-value (Supplementary Table S4). The top 10 bins from each module were shown in the form of a bar graph.

## Results and discussion

### Characterization of targeted endosperm fine tissues at early and middle storage phase

**Fig. 1:**
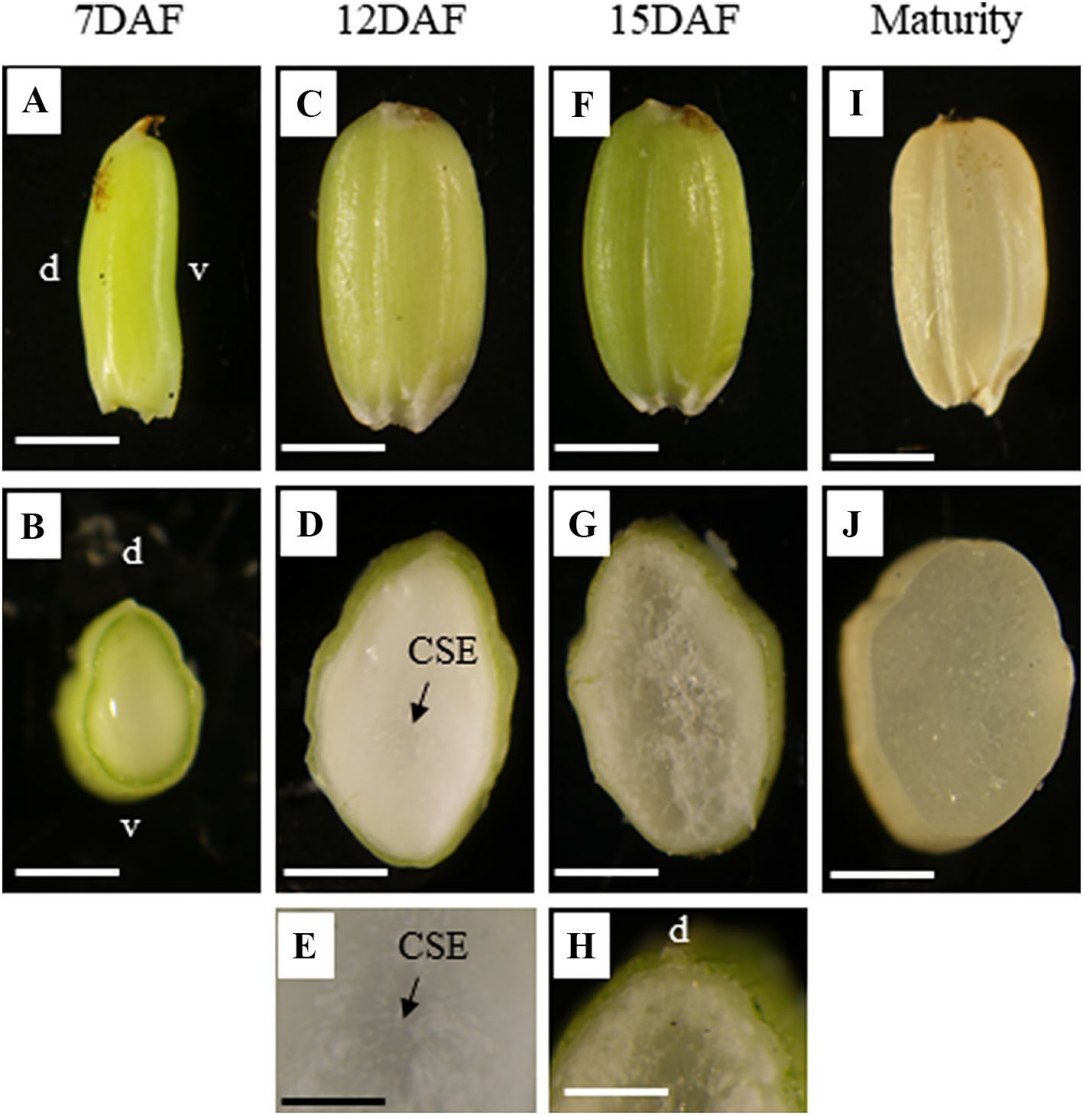
Development of caryopsis from 7DAF to maturity. DAF; days after flowering. **A**–**B** 7DAF; **C**–**E** 12DAF; **F**–**H** 15DAF; **I**–**J** maturity. **A**, **C**, **F**, **I**; Bars = 2 mm. **B**, **D**, **G**, **J**; Bars = 1 mm. E (magnified scale of center zone of starchy endosperm in **D**), **H** (magnified scale of dorsal zone of endosperm in **G**); Bars = 0.5 mm. *d* dorsal side, *v* ventral side, *CSE* center zone of starchy endosperm

In rice caryopsis development, 7DAF represents the initial storage phase and 12DAF is characterized as the most active storage phase in terms of dry matter accumulation (Ishimaru et al. 2003). At 7DAF, length of caryopsis was already determined (Ishimaru et al. 2003; Fig. 1A), and cellularization of endosperm is almost completed (Ishimaru et al. 2003) with ample water content (Ishimaru et al. 2009; Fig. 1B). At 12DAF, width of caryopsis was already determined (Ishimaru et al. 2003; Fig. 1C), with most zones of the endosperm looking milky white (Fig. 1D) except for the slight transparency at the center zone of the starchy endosperm (Fig. 1E). At 15DAF, the transparent zone spread to the medium layer of the endosperm (Fig. 1F, G, and H). At maturity, the entire zone of endosperm became transparent (Fig. 1I and J).

**Fig. 2:**
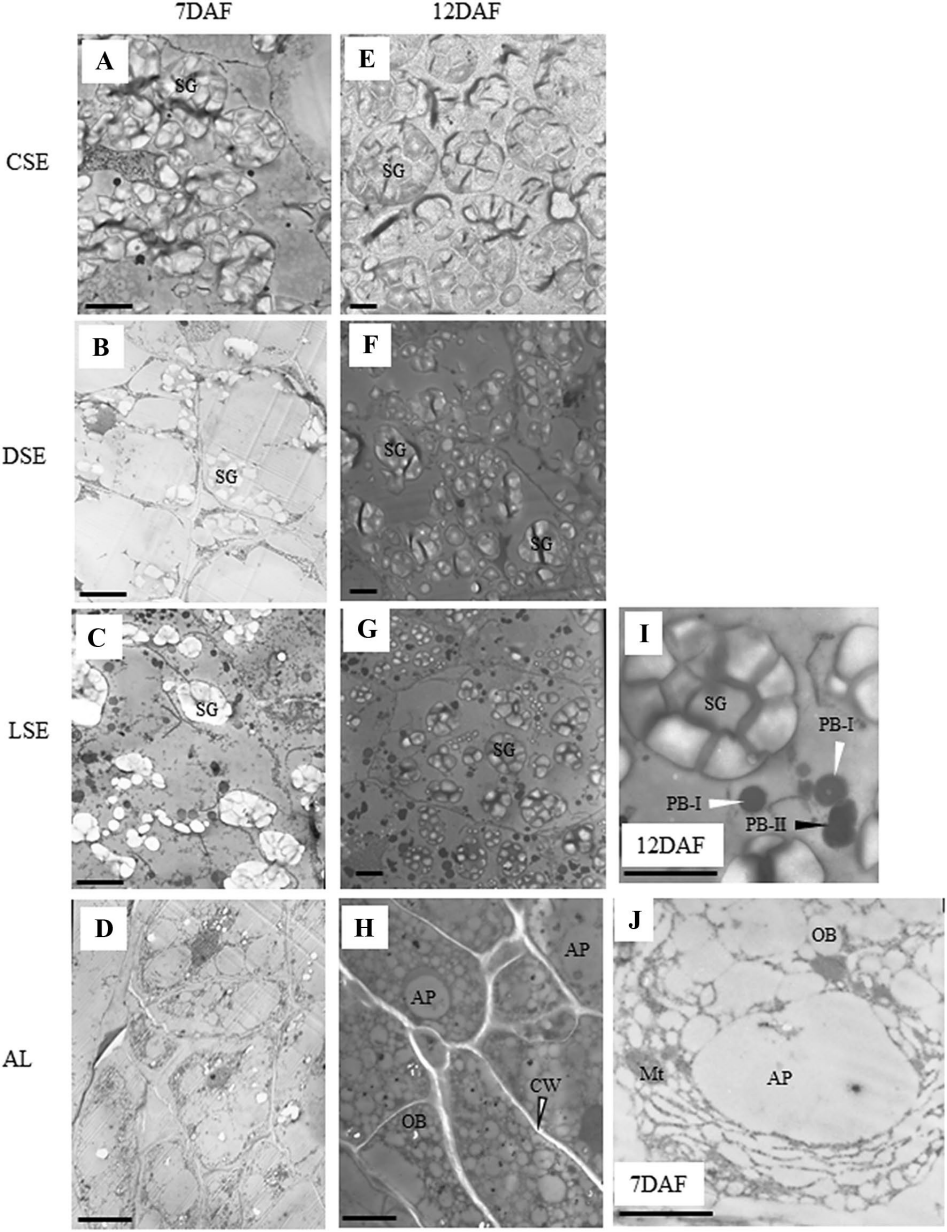
Transmission electron microscopic (TEM) observation at each endosperm zone. **A**–**D** 7DAF; **E**–**H** 12DAF; **I** magnified scale of LSE at 12DAF; **J** magnified scale of AL at 7DAF. **A** and **E** CSE; **B** and **F** DSE; **C**, **G**, and **I**, LSE; **D**, **H**, and **J**, AL. *AP* aleurone particles, *CW* cell wall, *Mt* mitochondria, *OB* oil body, *SG* starch granule, *PBI* protein body I, *PBII* protein body II. Bar = 5 μm

According to the TEM fine tissues in the starchy endosperm, the center zone was distinguished with the presence of starch granules (CSE, Fig. 2A and E); while the dorsal side (DSE, Fig. 2B and F) and lateral side (LSE, Fig. 2C and G) had less starch granules. Instead, AL contained aleurone particles, oil bodies, and mitochondria (Fig. 2H and J). Lipids are specifically localized at the aleurone cell layers (Ishimaru et al. 2015), and globulins are accumulated in those aleurone particles (Ogawa et al. 1979). On the other hand, starchy endosperm accumulates starch and storage proteins (prolamin and glutelin) with temporal and spatial differences. Starch granules were predominantly formed in the central zone of the endosperm tissues at 7DAF, and the accumulation of starch granules is extended in cells of DSE and LSE at 12DAF (Hoshikawa 1968). Protein bodies (PB) I and PBII are predominantly observed in LSE (Fig. 2I), but hardly observed in DSE and CSE (Fig. 2A, B, E, and F).

**Fig. 3:**
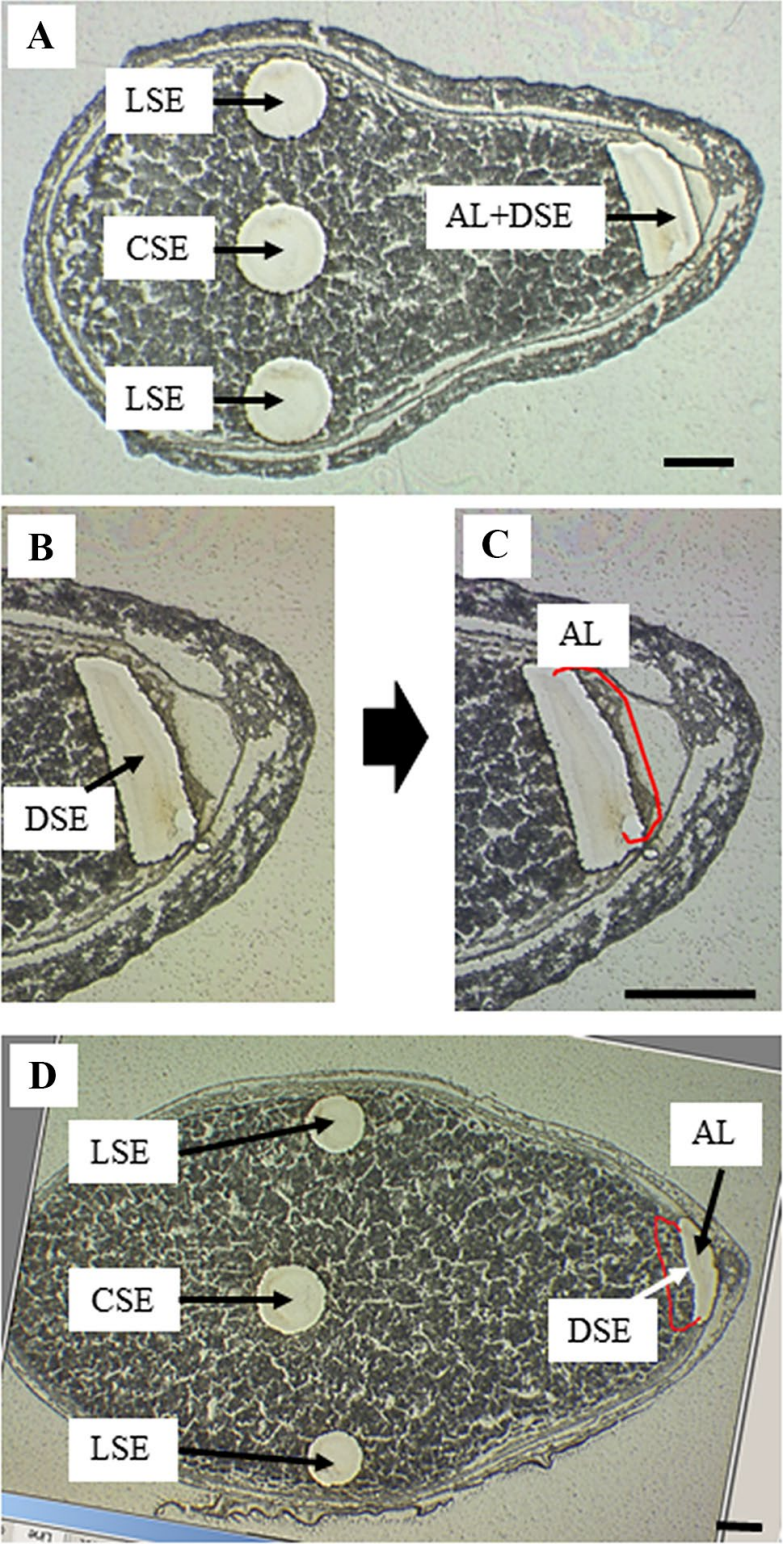
Isolation of fine endosperm tissues at dorsal aleurone layer (AL), center zone (CSE), dorsal zone (DSE), and lateral zone (LSE) of starchy endosperm at 7DAF and 12DAF by laser microdissection. **A** Isolation of fine endosperm tissues at 7DAF by LM. **B** and **C** magnified scale of dorsal side of endosperm to dissect DSE and AL zones. **D** Isolation of fine endosperm tissues at 12DAF by LM. Bars = 250 μm

In the present study, we targeted to identify the differential expression of genes in developing endosperm at 7DAF and 12DAF by isolating fine tissues of AL, CSE, DSE, and LSE with LM (Fig. 3A–D). The dynamic accumulations of storage compounds are spatially and temporally coincident with changes in water distribution in the developing rice endosperm (Horigane et al. 2001; Ishimaru et al. 2009). Overall microscopic observations showed distinct developmental and spatial accumulation patterns of starch granules, storage proteins (PBI, and PBII), and lipids among AL, CSE, DSE, and LSE.

### Fine-tuned co-regulons preferentially expressed in dorsal aleurone layers (AL) and distinct zones of starchy endosperm tissues (CSE, DSE and LSE) during the onset of cellularization and peak of storage phase

**Fig. 4:**
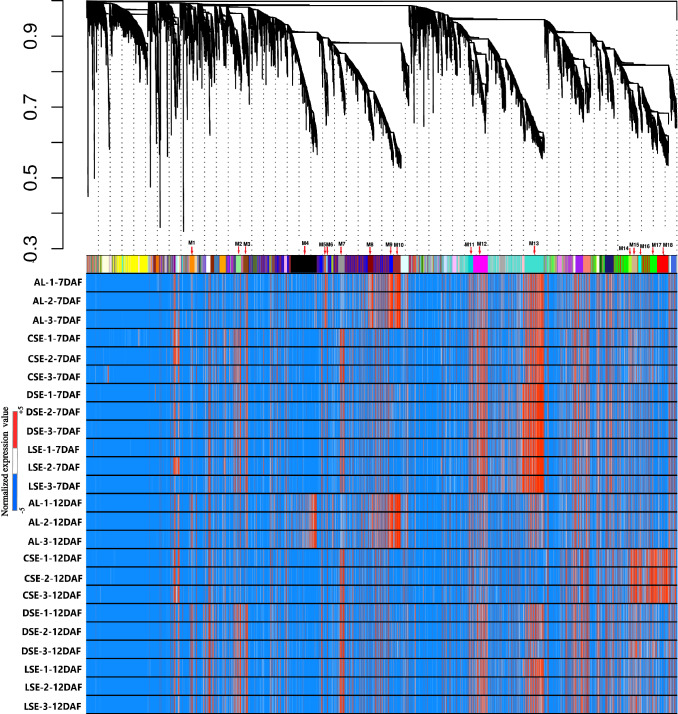
Gene dendrogram and corresponding heatmap of 60 distinct co-expressed modules. 18 modules depicted distinct fine-tuned co-expression patterns (co-regulons) of genes showing preferential or specific expression in distinct zones of fine tissues of AL, CSE, DSE, and LSE during 7 and 12DAF were selected. M1-orange, M2-lightgreen, M3-saddlebrown, M4-black, M5-salmon, M6-skyblue, M7-grey60, M8-darkred, M9-blue, M10-brown, M11-darkturquoise, M12-magenda, M13-turquoise, M14-greenyellow, M15-tan, M16-cyan, M17-green, and M18-red. AL-dorsal aleurone cells, CSE-center zone of starchy endosperm, DSE-dorsal zone of starchy endosperm, LSE-lateral zone of starchy endosperm. The number (1–3) stands for replicates derived from differentially expressed genes across AL, DSE, CSE and LSE tissues. Normalized expression value is indicated by color gradient

So far, the genome-wide analysis using mRNA/proteins extracted from whole grains revealed the detailed temporal changes in metabolic pathway (Gao et al. 2013; Xu et al. 2010) and identified seed-specific regulation of transcription factors (Sharma et al. 2012; Xue et al. 2012) during seed development. However, until now the heterogeneous functions of distinct endosperm compartments regulating distinct seed storage events have not been deciphered. The present study further provides the opportunity to derive gene regulatory networks to link the spatial storage processes in the developing rice endosperm. Based on weighted gene correlation network analysis using WGCNA method (Langfelder and Horvath 2008) followed by the topological overlap matrix (TOM) similarity algorithm, we identified 60 modules (M1-M60) (Fig. 4). Of them, 18 modules depicted distinct fine-tuned co-expression patterns (co-regulons) of genes showing preferential or specific expression in distinct zones of fine tissues of starchy endosperm (CSE, DSE, and LSE) and in dorsal aleurone layers (AL) during 7 and 12DAF (Fig. 4).

**Fig. 5:**
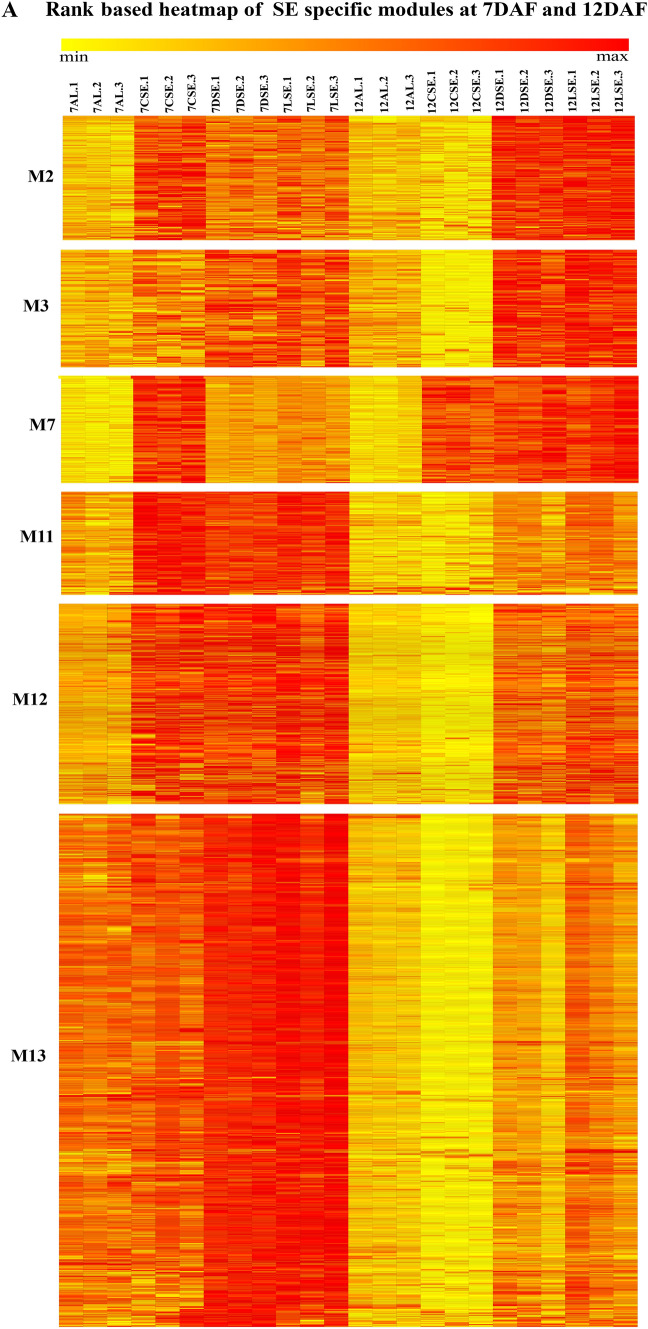

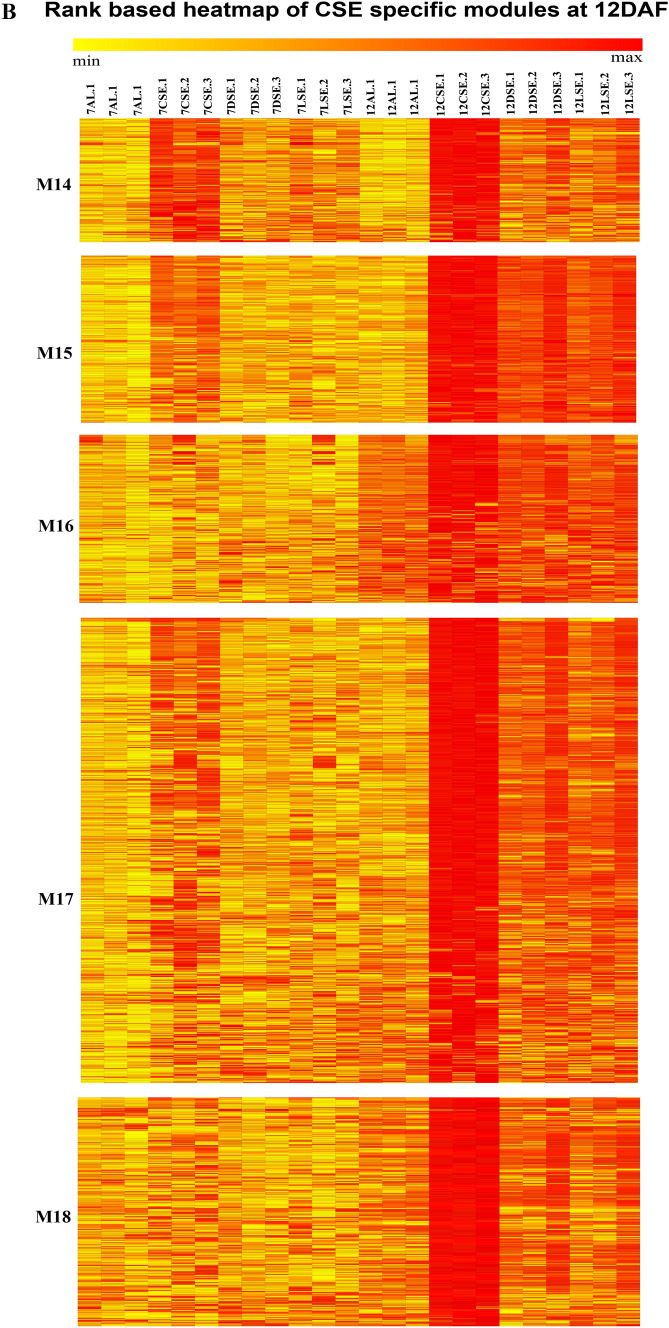

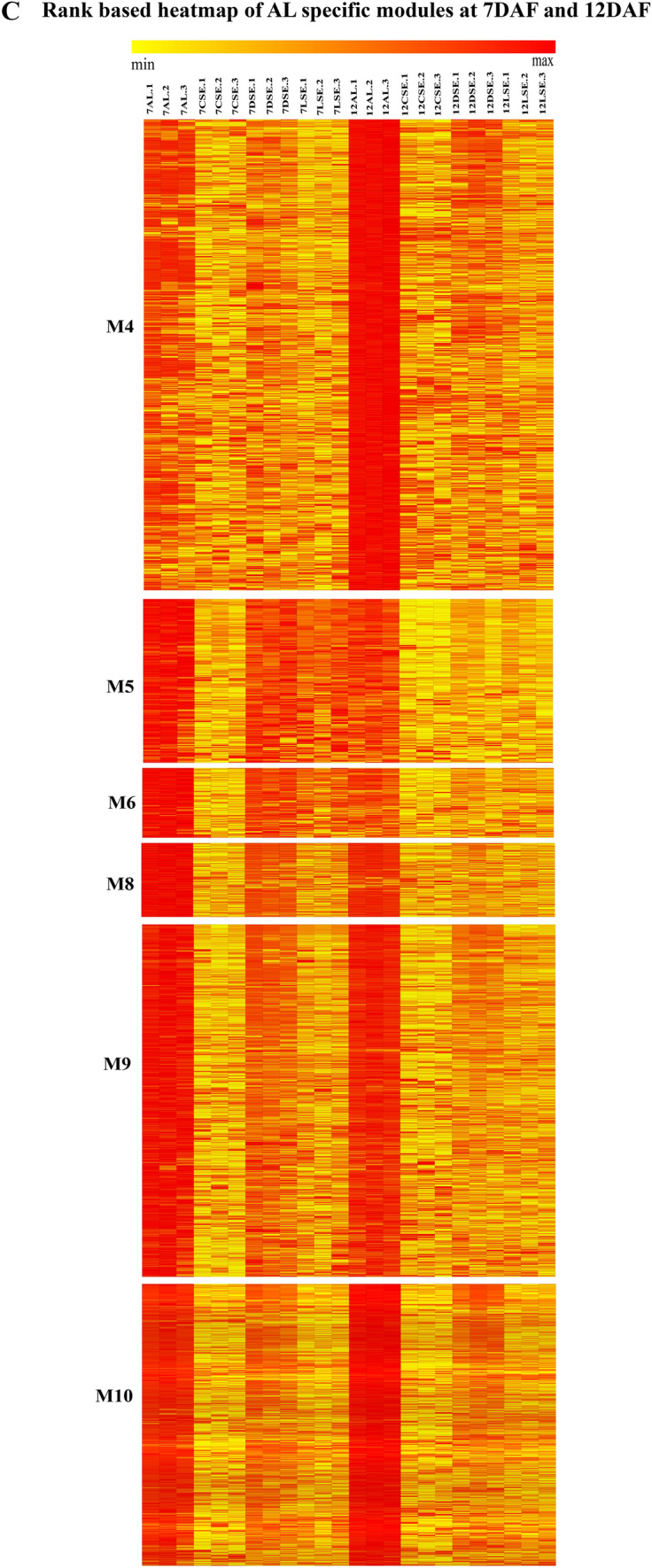
The heatmap of tissue specific modules at 7DAF and 12DAF. A-starchy endosperm (SE) tissues specifically expressed modules at 7DAF and 12DAF. B-center zone of starchy endosperm (CSE) tissues specifically expressed module at 12DAF. C-dorsal aleurone (AL) tissue specific module at 7DAF and 12DAF. Log_2_ expression value is indicated by color gradient

**Fig. 6:**
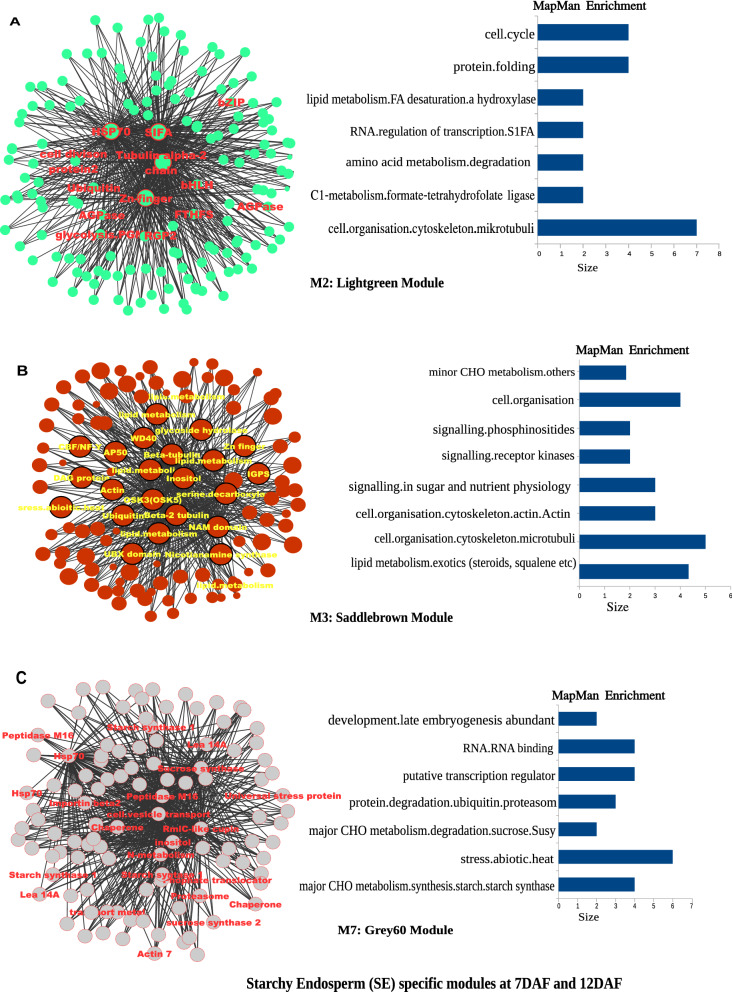

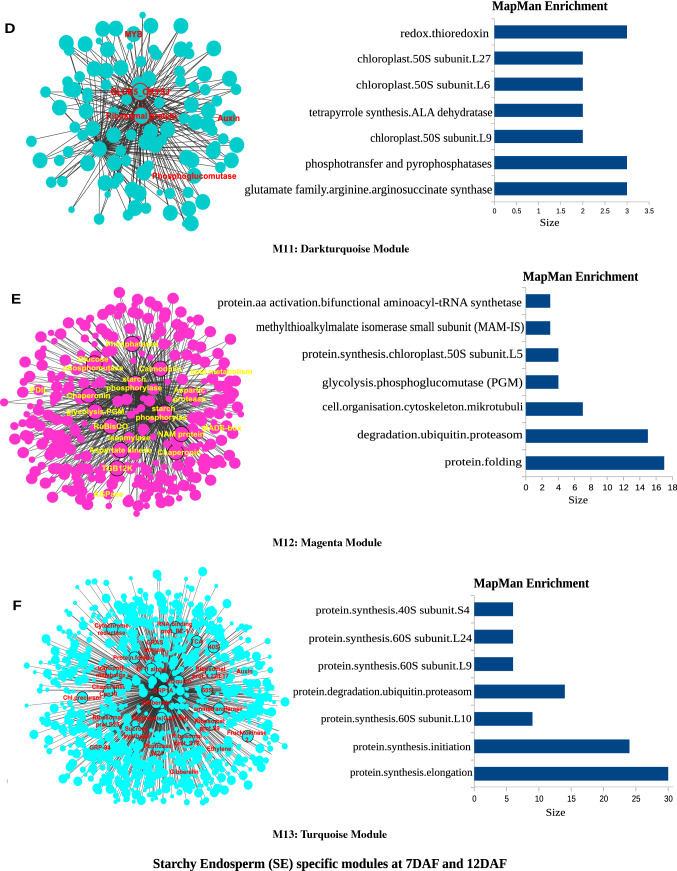
Gene regulatory network (left side) and MapMan enriched functions (right side) of starchy endosperm (SE) specific co-expressed modules at 7DAF and 12DAF. The hub genes (nodes) of six modules network namely, M2-lightgreen (**A**), M3-saddlebrown (**B**), M7-grey60 (**C**), M11-darkturquoise (**D**), M12-magenta (**E**), M13-turquoise (**F**) are highlighted in bordered circle. The top 10 bins of MapMan functional categories based on significant values are shown beside each module where the size of bars represents the number of bins

The heatmap of 18 modules showed the distinct fine-tuned patterns of expression, suggesting that complex spatial and temporal expression patterns exist in developing rice endosperm at early and middle storage phase. The modules M2, M3, M7, M11, M12, M13, M14, M15, M16, M17, and M18 are preferentially or even specifically expressed in starchy endosperm fractions in comparison to AL tissue (Fig. 5A, B, and C). Interestingly, there are finer differences observed within these modules (Table 1). For instance, M2 module preferentially expressed in CSE during 7 DAF and its expression patterns were found in LSE and DSE during 12 DAF (Fig. 5A, the top panel). The module M3 genes are expressed preferentially in LSE and DSE during 12 DAF (Fig. 5A, the second panel from the top). MapMan-based enriched functional categories showed that genes involved in cell organization, cytoskeleton, and microtubulin were enriched in M2 and M3 (Fig. 6A and B), suggesting that those genes have a role at first to complete cellularization in the CSE zone during 7 DAF and in DSE and LSE regions during 12 DAF. Hoshikawa (1967) found that rapid cell elongation in starchy endosperm occurs around the central zone spread to the outer zone of starchy endosperm after the onset of starch accumulation. Expression profile in M2 and M3 depicts fine spatial and temporal regulation in the cell framework for developing endosperm, which supports the histological study of Hoshikawa (1967). Modules M11, M12 and M13 are preferentially expressed in various endosperm fractions during 7 DAF and are least expressed in CSE during 12 DAF (Fig. 5A, bottom three panels). The co-expressed regulons of M11 imply amyloplast development pathway (Fig. 6D), M12 and M13 modules with glycolysis and protein biosynthesis machinery (Fig. 6E and F). The M7 module genes are preferentially expressed in CSE during 7 DAF and 12 DAF and as well in LSE and DSE during 12 DAF (Fig. 5A, the third panel from the top), and enriched for starch metabolism (Fig. 6C). The co-expressed modules M14, M15, M16, M17 and M18 are preferentially or even specifically expressed in CSE with subtle differences between those modules (Fig. 5B). Critical gene regulatory network for accumulation of starch and storage protein, but not the accumulation of lipid, is hypothesized to exist in these six modules (Fig. 6C, 7A–E). Although the modules M4, M5, M6, M8, M9, and M10 are preferentially expressed in AL, the individual modules show temporal variation between 7 and 12 DAF (Fig. 5C). These gene regulatory networks which are preferentially expressed in AL are enriched for redox, antioxidants, various transporters and triacylglycerol biosynthesis (Fig. 8A–F). In the following subsections, we infer the in-depth analyses by co-expression gene regulatory network and MapMan-based enriched functional categories of genes in relation to the tissue- and stage-specific storage compound accumulation in each endosperm zone.

**Table 1:** Characterization of expression pattern in 18 modules with tissue- and stage-specificity or preference

Module no	Module color	No. of genes	Key feature	Minor feature(s)
M1	Orange	70	Preferential in AL, DSE, and LSE at 12DAF	Preferential in AL at 7DAF
M2	Lightgreen	154	Preferential in starchy endosperm at 7DAF and 12DAF	Low in CSE at 12DAF
M3	Saddlebrown	113	Preferential in DSE and LSE at 7DAF	Very low in CSE at 12DAF
M4	Black	686	Preferential in AL at 12DAF	Relatively high in AL at 7DAF
M5	Salmon	157	Preferential in AL at 7DAF	Relatively high in DSE and LSE at 7DAF, and AL at 12DAF
M6	Skyblue	93	Preferential in AL at 7DAF	Relatively high in DSE at 7DAF and AL at 12DAF
M7	Grey60	107	Almost absent in AL both at 7DAF and 12DAF	Relatively high in starchy endosperm at 7DAF, high in starchy endosperm at 12DAF
M8	Darkred	143	Preferential in AL at 7DAF and 12DAF	Relatively high in DSE at 7DAF
M9	Blue	585	Preferential in AL at 7DAF and 12DAF	Relatively high in DSE at 7DAF
M10	Brown	931	Preferential in AL at 7DAF and 12DAF	Relatively high in starchy endosperm at 7 and 12DAF
M11	Darkturquoise	109	Preferential in starchy endosperm at 7DAF	Relatively high in other endosperm tissues
M12	Magenta	302	Preferential in starchy endosperm at 7DAF, and DSE and LSE at 12DAF	Almost absence in CSE at 12DAF
M13	Turquoise	926	Almost absent in CSE at 12DAF	High in all endosperm tissues at 7DAF
M14	Greenyellow	152	Preferential in CSE at 12DAF	Relatively high in starchy endosperm at 7DAF, and DSE and LSE at 12DAF
M15	Tan	170	Preferential in CSE at 12DAF	Relatively high in CSE at 7DAF, and DSE and LSE at 12DAF
M16	Cyan	236	Preferential in CSE at 12DAF	Relatively high in all endosperm tissues at 12DAF
M17	Green	556	Preferential in CSE at 12DAF	Relatively high in DSE and LSE at 12DAF
M18	Red	368	Specific in CSE at 12DAF	

Starchy endosperm stands for central starchy endosperm (CSE), dorsal starchy endosperm (DSE), and lateral starchy endosperm (LSE); dorsal aleurone cells (AL); Days after flowering (DAF); Module (M)

### Unraveling the potential regulators of starch metabolism

Gene regulatory network showed that three modules (M2, M7 and M12) were preferentially enriched with starch metabolism genes (Fig. 6A, C, and E). Genes co-expressed in module M2 includes starch metabolism genes (invertase, hexokinase, phosphoglucomutase and ADP-glucose pyropohosphorylase (AGPase) large subunit, cell division and cytoskeleton organization, protein folding (*HSP70*, *HSP20*), G-proteins (Rab11C, OsRac3, RGP2, RAB5B) and transcription factors (bHLH, Jumonji, homeobox, S1FA, C2C2(Zn) GATA), which are preferentially expressed in all tissue types of endosperm during 7 and 12 DAF (except CSE at 12DAF) (Supplementary Table S3). Genes co-expressed in M12 includes starch metabolism genes (hexokinase, monosaccharide transporters, phosphoglucomutase and AGPase small subunit, isoamylase, alpha 1,4-glucan phosphorylase, trehalose-phosphate/synthase), cell division and cytoskeleton organization, protein synthesis, protein targeting, protein degradation (26S ubiquitin proteasome, E2 ubiquitin, alanine protease, aspartine protease, serine protease), protein folding (chaperonins of 10KDa, 20KDa, 60KDa, *HSP70*, *HSP80, DnaJ*), G-proteins, calmodulins and transcription factors (MYB, bZIP, Zn-finger(CCHC)), which are preferentially expressed in all tissue types of endosperm during 7 and 12 DAF (except CSE at 12DAF) (Supplementary Table S3). The M7 co-regulons encode sucrose synthase 1 (*SUS1* and *SUS2*) and starch synthase 1 (*SSI*), glucose 6-phosphate (G6P) translocator from starch metabolism, depict distinct expression patterns with peak of expression in CSE during 7 DAF and expanding its spatial expression in LSE and DSE at 12 DAF. The starch accumulation is coincided with expression of two rate limiting genes such as sucrose cleavage genes (*SUS1* and *SUS2*) which produces UDP-glucose and AGPase large and small subunits involved in producing ADP-glucose, a precursor for the production of amylose and amylopectin chains in CSE during 7 DAF and in DSE and LSE during 12 DAF. In addition, we noticed expression of G6P translocator and ADP-glucose translocator in the starchy endosperm, suggesting that both pathways of cytosolic and plastidic production of ADP-glucose are possible in the heterogeneous endosperm tissue (Toyota et al. 2006). According to the TEM observation, active accumulation of starch was almost completed in CSE at 12DAF (Fig. 2E). The expression pattern of genes for starch metabolism in M2, M7 and M12 was spatially and temporally coincident with starch accumulation in the endosperm.

Toc34 and OEP75 encode a translocon that facilitates the translocation of polypeptide from cytosol to plastid at outer envelope membrane (Andrès and Agne 2010). Genes encoding chloroplastic outer envelope membrane protein (OEP75) (LOC_Os03g16440) and chloroplast protein import component Toc34 family protein (LOC_Os03g13730) were categorized into M7 (Supplementary Fig. S1). A plastid-localized heat shock protein (LOC_Os12g14070) in M7 regulates the protein import into amyloplast associated with Tic complex (Andrès and Agne 2010), thereby mutant lacking this gene shows the floury (chalky) phenotype with aberrant amyloplast development (Zhu et al. 2018; Tabassum et al. 2020). Regulation of starch accumulation by molecular chaperones in coordination with the Tic complex is noted in SE. Interestingly, a number of molecular chaperones HSP involved in protein folding were identified in four modules M2, M7, M12, and M13 (Fig. 6A, C, E, and F; Supplementary Fig. S2). The effect of other heat shock proteins categorized into M2, M7, M12, and M13 on the phenotype in starchy endosperm is still elusive.

**Fig. 7:**
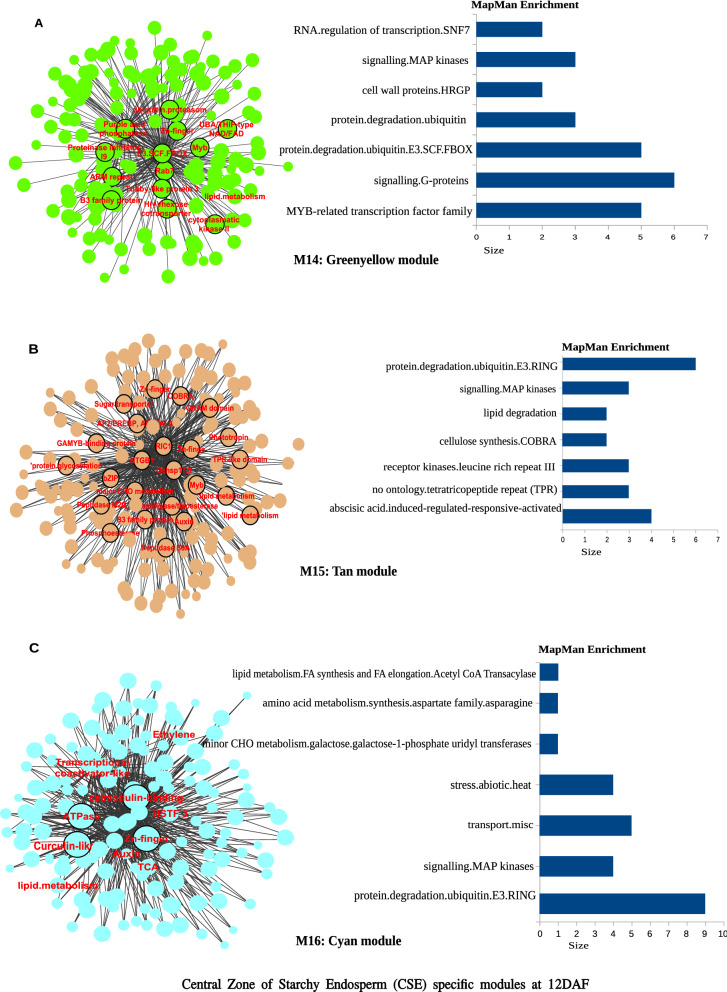

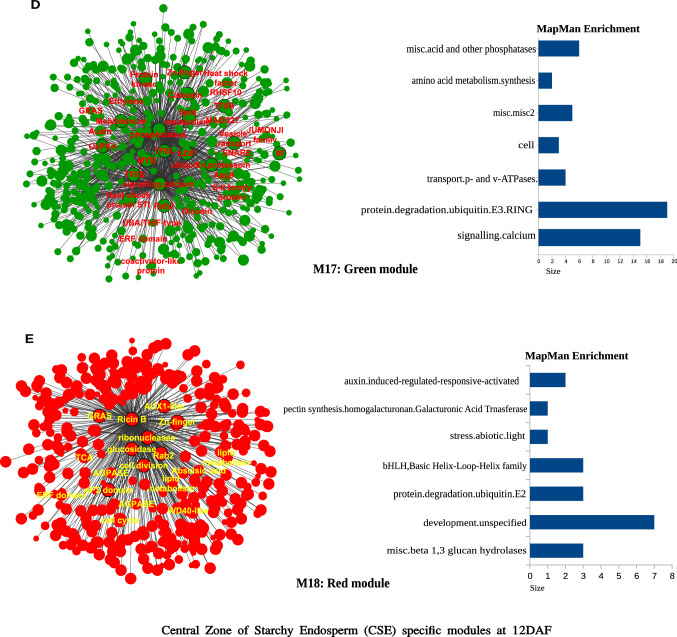
Gene regulatory network and MapMan enriched functions of central zone of starchy endosperm (CSE) specific co-expressed modules at 12DAF. The hub genes (nodes) of five modules, namely, M14-greenyellow (**A**), M15-tan (**B**), M16-cyan (**C**), M17-green (**D**), M18-red (**E**) are highlighted in bordered circle. The top 10 bins of Mapman enriched functional categories based on significant values are shown beside each module where the size of bars represents the number of bins

Co-expression gene regulatory network analysis showed that M14-18, which are characterized as high expression in CSE at 12DAF (Fig. 5B), involves lipid metabolism (Fig. 7A–E). Cells of CSE were almost occupied by starch granules at 12DAF (Fig. 2E), but not by lipids (Ishimaru et al. 2015). In fact, specific expression of CDP-alcohol phosphatidyltransferase (LOC_Os03g17520), glycerophosphoryl diester phosphodiesterase (LOC_Os02g37590), glycosyl transferase (LOC_Os03g15840),　acyl-CoA synthetase (LOC_Os01g48910), MGDG synthase type A (LOC_Os02g55910), phosphatidate cytidylyltransferase (LOC_Os01g55360), coclaurine N-methyltransferase (LOC_Os06g37610), which are involved in glycerolipid metabolism, phospholipid biosynthesis, glycerophospholipid metabolism, was observed in CSE at 12DAF (Supplementary Fig. S3). Genes for lipid metabolism showing specific expression in CSE at 12DAF (Supplementary Fig. S3) seemed to be quite different from those for lipid metabolism in AL (Supplementary Fig. S4). Recent genetic study with floury shrunken endosperm1 (*fse1*) revealed the involvement of phospholipid metabolism in amyloplast development in rice (Long et al. 2018). The *fse1* mutant results in white-cored type of mild chalk phenotype (Long et al. 2018), suggesting a role of phospholipid biosynthesis in the amyloplast development particularly in the center zone of starchy endosperm (CSE). Zhou et al. (2016) proposed a model that enhancement of lipid metabolism and amylose biosynthesis pathway in the developing endosperm is essential to increase the resistant starch (RS) type five content. RS type five consists of amylose–lipid complexes (Raigond et al. 2015) contributes to glucose homeostasis, which is beneficial to counter type II diabetes because of the restriction of starch granules to swelling during cooking and thus contributes to slower digestion. RS content is positively correlated with amylose content in the rice grains (Yang et al. 2006). Our LM-based expression analysis revealed that maintained high expression of *GBSSI* (LOC_Os06g04200), which is responsible for amylose biosynthesis (Itoh et al. 2003), in CSE at 12DAF (Supplementary Fig. S5). Amylose content is highest in CSE zone, followed by medium levels in DSE and LSE zones, and lowest in peripheral zones (AL) in the rice endosperm (Itani et al. 2002). Simultaneous high expression level of *GBSSI* (Supplementary Fig. S5) and specific lipid metabolism (Supplementary Fig. S3) occurred in CSE at the middle storage phase. The present LM-based expression data in CSE at 12DAF provided clues to elucidate the genes which are involved in the resistant starch biosynthetic pathway in rice.

### Starch accumulation patterns in endosperm precedes non-degenerative programmed cell death (PCD) under desiccation tolerant mechanism

Kobayashi et al. (2013) demonstrated that PCD initiated in the CSE zone at middle storage phase, then spread to the peripheral zone of starchy endosperm. We hypothesized that our LM-based expression analysis could reveal the genes that played a critical role in the onset of PCD in the CSE zone at the middle storage phase. MapMan-based enriched functional categories of genes indicated the ubiquitin-dependent protein degradation in M14, M15, M16, M17, and M18 preferentially upregulated in CSE at 12DAF (Fig. 7A–E). PCD is largely used to describe the process of apoptosis and autophagy (Reape et al. 2008), and these genes are preferentially expressed in modules M14-18. Autophagy protein 8 (LOC_Os04g53240) and Autophagy protein Apg9 family protein (LOC_Os03g14380) were found in M15 and M17, respectively (Supplementary Fig. S6). Furthermore, autophagy-related proteins, a UBA/THIF-type NAD/FAD binding fold domain containing protein (LOC_Os01g42850) and two WD40-like domain containing protein (LOC_Os01g57720 and LOC_Os05g33610) were found to be expressed in M17 and M18, respectively with very high expression only in CSE at 12DAF (Supplementary Fig. S6). These results indicate a possibility that PCD through autophagy would occur in CSE at the middle stage. Our LM-based tissue specific expression analysis supports PCD events histologically demonstrated by Kobayashi et al. (2013). The CSE zone started transparency (Fig. 1D and E) after the completion of starch accumulation there at middle storage phase (Fig. 2E), meaning the drastic reduction in water content in CSE zone during the middle storage phase (Horigane et al. 2001; Ishimaru et al. 2009). It could be hypothesized that the CSE zone has a specific biological strategy to adapt the dehydration process toward maturation.

The genes involved in abiotic stress response were preferentially enriched in modules M14-M18. These includes, transcription factors, C-myb-like transcription factor (LOC_Os01g62410, designated as OsMYB3R-2, Dai et al. 2007), MADS27 transcription factor (LOC_Os02g36924, designated as OsMADS27, Chen et al. 2018) (Supplementary Fig. S7) are suggested to regulate abiotic stress tolerance in rice plants. We found heat shock factor protein 3 (LOC_Os09g35790, HSF 3) and heat stress transcription factor Spl7 (LOC_Os04g48030) as potential candidates for abiotic stress tolerance transcription factor (Supplementary Fig. S7). Specific expression of calcium-dependent protein kinase (LOC_Os03g03660, OsCPK7 or OsCDPK11), two calcium-binding EF-hand domain containing proteins (LOC_Os05g31620 and LOC_Os09g24580), Calmodulin 2/3/5 (LOC_Os01g72100) in M18 were observed in CSE at 12DAF (Supplementary Fig. S8). MapMan-based enriched functional categories of genes indicated the critical involvement of calcium-mediated signaling in M17 (Fig. 7D) with specific expression of two calcium-dependent protein kinase (LOC_Os03g03660 and LOC_Os04g49510) and calcium-binding EF-hand domain containing protein (LOC_Os01g72080) in CSE at 12DAF (Supplementary Fig. S8). These results suggest the calcium-dependent signal transduction pathway has an important role in CSE at 12DAF. Innermost layers corresponding to the CSE zone contained 45% calcium, when compared to the entire rice grains, thereby it is suggested that calcium is essential for cell development in the inner zone of endosperm during grain filling (Itani et al. 2002). We postulate that calcium-calmodulin-dependent protein kinase cascade has a crucial role in signal transduction during the dehydration process that started in CSE at middle storage phase. Very specifically, two carbonic anhydrase (CA) (LOC_Os01g45274 and LOC_Os09g28150 in M18), an enzyme which has main role in supplying CO_2_ for carbon fixation by Rubisco by catalyzing the reversible hydration of CO_2_ in leaves, were specifically expressed in CSE at 12DAF (Supplementary Fig. S9). LOC_Os09g28150 is the α-type CA, whereas LOC_Os01g45274 is the β (chloroplast)-type CA. In the leaves of rapeseeds, Wang et al. (2016) suggested the involvement of phosphorylation of β-type CA for adaptation to drought stress. It is likely that β-type carbonic anhydrase may play an essential role in adaptation to the dehydration process in the amyloplast after completion of starch accumulation in CSE. Involvement of α-type carbonic anhydrase in dehydration adaptation remains an open question.

Co-expression gene regulatory network analysis indicated the involvement of plant hormones such as auxin (M15, M16, M17, and M18), abscisic acid (M18), ethylene (M16 and M17) (Fig. 7A–E). Those plant hormones may influence the abiotic stress tolerance during cellular dehydration process in CSE after the middle stage, possibly in coordination with transcription factors and genes related to stress tolerance described above. On the other hand, CSE predominantly accumulates starch (Fig. 2E). CSE is the first endosperm tissue to start dehydration (Fig. 1D and E) while other SE tissues are in the peak of starch accumulation. An abiotic stress tolerance mechanism in CSE at middle stage would contribute to preserving the starch granules as a reserve for the energy source at germination of the next generation.

### Lateral starchy endosperm (LSE) gene regulatory network infers ongoing storage protein accumulation

Predominant localization of PBI (prolamin) and PBII (glutelin) in the LSE zone indicated large amounts of storage proteins there, according to the TEM observation (Fig. 2C, G, and I). In spite of such predominant localization of PBI and PBII in LSE zone, only two genes for glutelin were found in M11 and M15 (Supplementary Fig. S10), whereas no gene for prolamin was categorized into any module (Supplementary Table S3). These results suggest that predominant localization of PBI and PBII in the LSE zone is determined by other regulators. Genes categorized into M11 and M13 showed that expression was almost absent in CSE at 12DAF although it was high in all endosperm tissues at 7DAF (Fig. 4; Table 1), suggesting that M11 and M13 would contain the important genes for protein synthesis and/or initial amyloplast development. Co-expression analysis of gene regulatory networks revealed that a transcriptional factor, Myb, DNA-binding domain containing protein (LOC_Os02g02370) was categorized into M11 (Fig. 6D) with Glu-B5 (LOC_Os02g14600). Glu-B5 mutant, W3660, drastically reduces glutelin content in the rice grain (Wang et al. 2005). M11 may possess the genes that are involved in synthesis of glutelin. Co-expression analysis of gene regulatory networks in M13 suggests the involvement of GRP94, plant hormones such as gibberellin, ethylene, and auxin (Fig. 6F). GRP94 (LOC_Os06g50300) is a molecular chaperone localized in endoplasmic reticulum (ER). Ishimaru et al. (2019) reported that expression of the genes for ER-localized molecular chaperones is thermo-sensitive, suggesting the regulation of prolamin content and initial amyloplast development in rice grain. Expression pattern of GRP94 showed that expression level was preferentially high in starchy endosperm (CSE, LSE, DSE) at 7DAF, then expression level maintained high at LSE at 12DAF (Supplementary Fig. S10). In addition, M13 possesses the glutelin A-1 storage protein (LOC_Os02g25860), which built diverse network to the neighboring genes including gibberellin-induced MYB transcriptional factor (LOC_Os03g38210) (Fig. 6F; Supplementary Table S3). Co-regulons of M13 module encodes the expression of various sucrose cleaving enzymes (invertases and sucrose synthase), trigger of glycolysis, mitochondrial electron transport, TCA and amino acid metabolic pathways (Fig. 6F). There also found two gibberellin-regulated 60S ribosomal protein L9 (LOC_Os09g31180 and LOC_Os02g01332) in M13 (Fig. 6F). M13 may possess the genes that are involved in synthesis of prolamin and glutelin as well as the genes that are involved in starch accumulation. Whether gibberelin influences the protein synthesis and/or initial amyloplast development through the ribosomal protein needs to be further investigated.

### Aleurone-specific gene regulatory networks characterized to be involved in oil deposition, assimilate transport, stress tolerant and defense mechanism, TCA cycle and reactive oxygen species scavenging

**Fig. 8:**
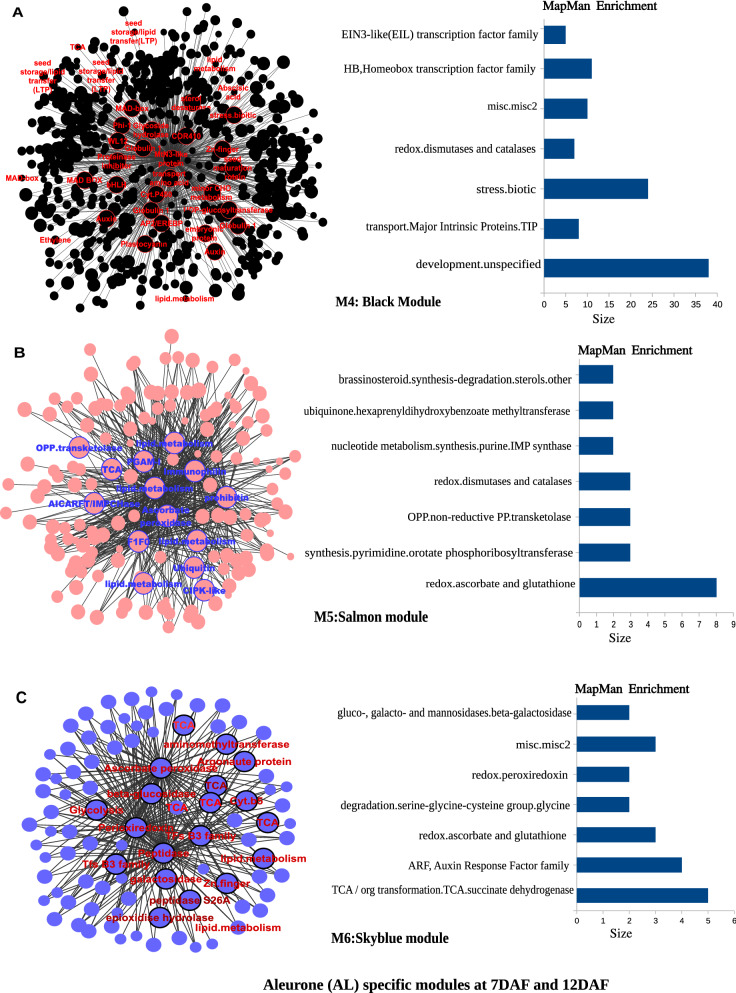

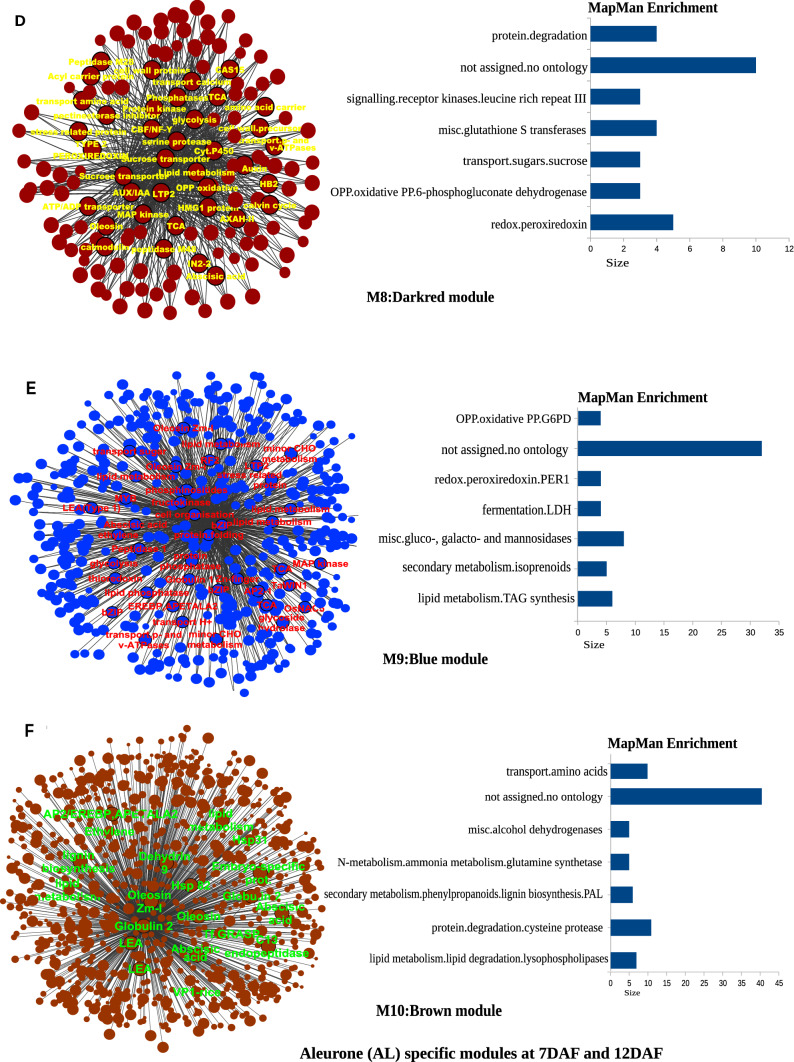
Gene regulatory network and MapMan Enriched functions of dorsal aleurone cells (AL) specific co-expressed modules at 7DAF and 12DAF. The hub genes (nodes) of six modules network namely, M4-black (**A**), M5-salmon (**B**), M6-skyblue (**C**), M8-darkred (**D**), M9-blue (**E**), M10-brown (**F**) are highlighted in bordered circle. The top 10 bins of MapMan functional categories based on significant values are shown beside each module where the size of bars represents the number of bins

Dorsal aleurone layers (AL) contain large amounts of lipids as oil bodies (Fig. 2H, J; Ishimaru et al. 2015). AL plays a specific role in uptake of sucrose and amino acids from maternal tissues into developing endosperm until the late storage phase (Opakra and Gates 1981). Cell morphology in AL was quite different from that in other starchy endosperm tissues, especially in terms of storage compounds (Fig. 2A–J). Elucidating the molecular mechanisms in the development of AL will be useful to understand assimilate transport, positional cell fate and morphogenesis, and oil deposition. Co-expression analysis showed that six modules (M4, M5, M6, M8, M9, and M10) possessed the AL-specific expression (Fig. 5C). Co-expression analysis of gene regulatory networks indicated the involvement of genes for lipid metabolism in all six modules (Fig. 8A–F), suggesting that those genes are involved in the biological cellular event in AL for oil deposition. Evidence suggests that specific expression of NADH-dependent enoyl-ACP reductase (LOC_Os08g23810 in M5), Acetyl-coenzyme A carboxylase (LOC_Os05g22940 in M6), dihydrolipoamide S-acetyltransferase (LOC_Os12g08170 in M5; LOC_Os08g33440 in M8), Pyruvate dehydrogenase E1 beta subunit (LOC_Os03g44300 in M9), Pyruvate kinase isozyme G (LOC_Os01g47080 in M9), Long-chain-fatty-acid–CoA ligase 4 (LOC_Os11g06880 in M9), etc. in AL reinforces the participation of those genes in fatty acid biosynthesis and chain elongation in AL (Supplementary Fig. S4). Co-expression analysis of gene regulatory networks and MapMan-based enriched functional categories of genes also indicated the involvement of genes for TCA cycle in M4, M5, M6, M8, and M9 (Fig. 8A–E). Ishimaru et al. (2015) previously demonstrated with LM that AL predominantly expresses the genes for TCA cycle and oxidative phosphorylation in the presence of oxygen and large numbers of mitochondria at early storage phase. Expression of genes for TCA cycle in M4, M5, M6, M8, and M9 (Fig. 8A–E) contributes to maintaining the redox status in AL to supply ATP for oil deposition through aerobic respiration during ripening.

MapMan-based enriched functional categories of genes indicated the unspecified development in M4 (Fig. 8A) with preferential expression of genes in AL at 12DAF (Fig. 5C, the top panel), suggesting that genes categorized into M4 have a particular role(s) in maintaining homeostasis at the middle storage phase. Co-expression analysis of gene regulatory networks indicated the genes for globulin were linked with the number of neighboring genes in M4 (Fig. 8A) as well as M9 and M10 (Fig. 8E and F). The genes are considered to be involved in the synthesis of 7S globulin, which is specifically accumulated in aleurone cells (Shewry 1995). In soybean and wheat, it is known that 7S globulin is not a storage protein, but a multi-functional protein with abiotic stress responses (Hirano et al. 1992; Omi et al. 1996) and endoxylanase inhibitor for the defense mechanism from pathogenic bacteria and fungi (Fierens et al. 2003; Yoshizawa et al. 2011). AL accumulates 7S globulin possibly for the abiotic stress response and defense mechanism to pathogens in rice. Dehydrin and late embryogenesis abundant (LEA) II protein, whose genes were categorized into M10 in gene regulatory network (Fig. 8F), also confer the desiccation tolerance to seeds during maturation in cereals (See review, Kosavá et al. 2014). M4, M9, and M10 possess many AL-specific transcription factors related to abiotic stress responses and pathogen defense (Supplementary Fig. S11). A transcription factor, MADS-box domain containing protein (LOC_Os08g41950, designated as OsMADS7, Zhang et al. 2018), a heat shock transcription factor 29 (LOC_Os01g53220, designated as OsHsfC1b, Schmidt et al. 2012), a transcription factor MYBS3 (LOC_Os05g37060, designated as MID1, Guo et al. 2016), and a heat shock transcription factor 31 (LOC_Os01g39020, designated as OsHsfA7, Liu et al. 2013), are suggested to regulate abiotic stress tolerance in rice plants. On the other hands, WRKY transcription factor 71 (LOC_Os02g08440, designated as OsWRKY71, Liu et al. 2007), WRKY transcription factor 51 (LOC_Os04g21950, designated as OsWRKY51, Hwang et al. 2016), and Transcription factor jumonji, JmjN domain containing protein (LOC_Os01g67970, designated as JMJ705, Li et al. 2013) are suggested to regulate pathogen defense signaling pathway in rice plants in crosstalk with plant hormones. A set of different genes specifically expressed in AL suggests that AL may have a different stress adaptation strategy including desiccation tolerance from CSE during maturation.

In the gene regulatory network, M4, M8, M9, and M10 possessed various plant hormones such as abscisic acid, ethylene, auxin (Fig. 8A, D, E, and F), and ethylene-responsive transcription factor-like protein, AP2/EREBP, suggesting the relation of plant hormones in induction of genes involved in the stress tolerance and defense mechanism in AL. Among AP2/EREBP genes, the AP2 domain containing protein RAP2.6 (LOC_Os03g08470) was specifically expressed in AL at 12DAF in M4 (Supplementary Fig. S12). Recently, Xiong et al. (2018) reported that alleles of LOC_Os03g08470, designated as *OsLG3*, from upland rice confers the drought tolerance in rice by inducing reactive oxygen species scavenging. According to MapMan-based enriched functional categories, genes involved in redox, ascorbate–glutathione cycle, detoxifies H_2_O_2_, were prominent in module M5 (Fig. 8B) and M6 (Fig. 8C). AL functions as a main route of assimilates into developing endosperm (Oparka and Gates 1981), thereby AL must be active until the late storage phase (Fig. 1G and H) by maintaining moisture content at outer layers of endosperm (Horigane et al. 2001; Ishimaru et al. 2009). There is a possibility that an AP2/EREBP gene, *OsLG3*, activates the genes involved in redox, ascorbate–glutathione cycle, contributing to the maintenance of cell activity and function of AL until maturation. It should be noted, however, AP2/EREBP protein plays diverse roles in biological cellular processes. In the developing endosperm of barley, AP2/EREBP gene is highly expressed in developing endosperm transfer cells (Thiel et al. 2008), which corresponds to AL in the developing rice endosperm of this study. Because of influences of AP2/EREBP protein on seed size, grain weight, and storage compound accumulation in Arabidopsis (Jofuku et al. 2005; Ohto et al. 2005), Thiel et al. (2008) speculated that AP2/EREBP that is highly expressed in endosperm transfer cells has similar effect on barley seed development as demonstrated by Arabidopsis. Whether AP2/EREBP family proteins categorized into M4, M9, and M10 (Supplementary Table S3) have a similar influence on seed characters in rice as demonstrated by Arabidopsis seeds or not need to be further investigated.

Arabidopsis leafy cotyledon 1 (LEC1) is an embryo-specific NUCLEAR FACTOE Y (NF-Y) transcription factor that regulates embryogenesis (Lothan et al. 1998). In rice, OsNF-YB1 (LOC_Os02g49410) homologous to LEC1 is specifically expressed in aleurone cells of developing endosperm (Sun et al. 2014) and regulates the grain filling by controlling a sucrose transporter, *OsSUT1* (LOC_Os03g07480, Hirose et al. 1997) interacting with an ERF transcription factor Os#ERF115 (LOC_Os08g41030) (Bai et al. 2016; Xu et al. 2016). Our LM-based expression analysis also confirmed the aleurone-specific expression of OsNF-YB1, OsERF115, and OsSUT1 in M8, M9, and M8, respectively (Supplementary Fig. S13). Other transporters such as monosaccharide, amino acid, ion, inorganic transporters were found in M4, M9, and M10 (Supplementary Fig. S13), supporting the essential role of AL for unloading of nutrients into developing endosperm. Transcription factors in four modules (M4, M8, M9, M10), which showed specific or preferential expression in AL (Fig. 5C), would structure the cascade with aleurone cell morphogenesis and transport-related genes during grain filling.

## Conclusion

Our LM-based tissue- and stage-specific expression analysis offers specific molecular physiological insights into the ongoing distinct storage processes occurring spatially in distinct fractions of developing endosperm. Further in-depth analysis by co-expression gene regulatory network and MapMan-based enriched functional categories of genes implied tissue- and stage-specific biological cellular events in the developing rice endosperm as shown in Table 2. The development of cell framework was assumed to be active in all starchy endosperm tissues at early storage phase although those events ceased in CSE at middle stage after the decline of starch accumulation. Starch metabolism, facilitation of the polypeptides’ translocation to amyloplast, protein holding by molecular chaperone for starch synthesis, and storage protein accumulation were assumed to be the key biological cellular events active in all starchy endosperm both at early and middle storage phases. Very interestingly, ubiquitin-dependent protein degradation, PCD through autophagy, phospholipid and glycolipid metabolism along with amylose synthesis, and dehydration stress response were assumed to be the specific biological cellular events predominant in CSE at the middle storage stage. Transcription factors, plant hormone-inducible proteins identified as co-regulators which are operative in starch metabolism, its functions need to be established in future studies through genome editing. Oil deposition through lipid metabolism, energy production through TCA cycle, redox and ROS scavenging through ascorbate–glutathione cycle, abiotic stress response and defense mechanism from pathogens, aleurone cell morphogenesis, and assimilate transport were assumed to be the key biological cellular events in AL both at early and middle storage phases. In conclusion, our LM-based tissue- and stage-specific transcriptome analysis provided novel insight into the molecular physiological mechanisms of rice endosperm development from early to middle storage phase.

**Table 2:** A hypothesis of tissue- and stage-specific biological cellular events in the developing rice endosperm based on the gene expression profile of distinct 18 modules

Endosperm tissue(s)	Key biological cellular events (modules involved)	
	Early storage phase (7DAF)	Middle storage phase (12DAF)
AL	Oil deposition through lipid metabolism (M4, M5, M6, M8, M9, M10) Energy production through TCA cycle (M4, M5, M6, M8, M9) Redox and ROS scavenging though ascorbate–glutathione cycle (M5, M6) Abiotic stress responses and defense mechanism from pathogens (M4, M9, M10) Cell morphogenesis and assimilate transport (M4, M8, M9, M10)	
Starchy endosperm (CSE, DSE, LSE)	Development of cell framework (M2, M3)^a^	
	Starch metabolism, facilitation of the polypeptide translocation to amyloplast, protein holding by molecular chaperone for starch synthesis (M2, M7, M12, M13) Storage protein accumulation (M11, M13)^b^	
CSE		Ubiquitin-dependent protein degradation (M14, M15, M16, M17, M18) PCD through autophagy (M15, M17, M18) Lipid metabolism (M14, M15, M16, M17, M18) Dehydration stress response (M17, M18)

Starchy endosperm stands for central starchy endosperm (CSE), dorsal starchy endosperm (DSE), and lateral starchy endosperm (LSE); dorsal aleurone cells (AL). Days after flowering (DAF); Module (M); programmed cell death (PCD); reactive oxygen species (ROS); tri carboxylic acid (TCA)

^a^Continue to middle storage phase in DSE and LSE

^b^Almost absent in CSE at middle storage phase

## Supplementary Information

Below is the link to the electronic supplementary material.

Supplementary file1 (TIF 839 kb) Supplementary Figure S1 Expression pattern of genes for SE-preferential Toc34 and OEP75. The gradient of min to max expression pattern follows yellow-darkorange color mode. For sample designation in each column, 7AL.1 in the column stands for 7DAF(7)-Dorsal aleurone layers(AL)-Replicates1(1). Abbreviations for stages and tissues follow Figure 1 and Figure 3, respectively. For gene designation in each line, M7 represents module7, LOC_Os03g16440 represents for MSU7, and ‘29.3.3_protein.targeting.chloroplast’ represents gene ontology (biological process) estimated by MapMan.

Supplementary file2 (TIF 1931 kb)

Supplementary file3 (TIF 1128 kb)

Supplementary file4 (TIF 1006 kb)

Supplementary file5 (TIF 662 kb)

Supplementary file6 (TIF 772 kb)

Supplementary file7 (TIF 1983 kb)

Supplementary file8 (TIF 1395 kb)

Supplementary file9 (TIF 843 kb)

Supplementary file10 (TIF 783 kb)

Supplementary file11 (TIF 955 kb)

Supplementary file12 (TIF 1461 kb)

Supplementary file13 (TIF 1721 kb)

Supplementary file14 (XLSX 3714 kb)

Supplementary file15 (XLS 7557 kb)

Supplementary file16 (XLSX 3863 kb)

Supplementary file17 (XLS 335 kb)

## Data Availability

All microarray data can be accessed from the GSE 181762 NCBI GEO repository.
