# Supplementary figures and images for: Laser microdissection transcriptome data derived gene regulatory networks of developing rice endosperm revealed tissue- and stage-specific regulators modulating starch metabolism
Source: Plant Mol Biol. 2022 Jan 31;108(4-5):443–67. doi: 10.1007/s11103-021-01225-w (PMC8894313; doi:10.1007/s11103-021-01225-w)

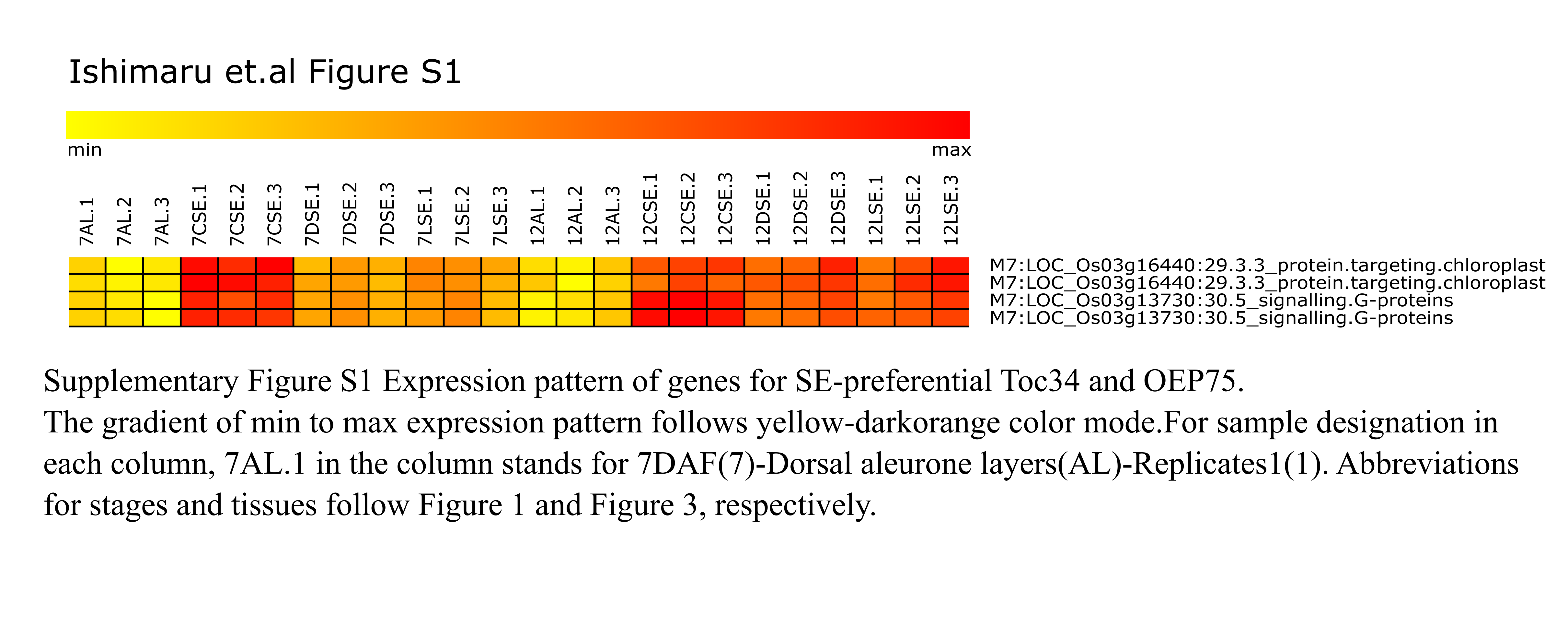

Supplement: Supplementary file 1 — Supplementary file1 (TIF 839 kb) Supplementary Figure S1 Expression pattern of genes for SE-preferential Toc34 and OEP75. The gradient of min to max expression pattern follows yellow-darkorange color mode. For sample designation in each column, 7AL.1 in the column stands for 7DAF(7)-Dorsal aleurone layers(AL)-Replicates1(1). Abbreviations for stages and tissues follow Figure 1 and Figure 3, respectively. For gene designation in each line, M7 represents module7, LOC_Os03g16440 represents for MSU7, and ‘29.3.3_protein.targeting.chloroplast’ represents gene ontology (biological process) estimated by MapMan. [file 11103_2021_1225_MOESM1_ESM.tif]

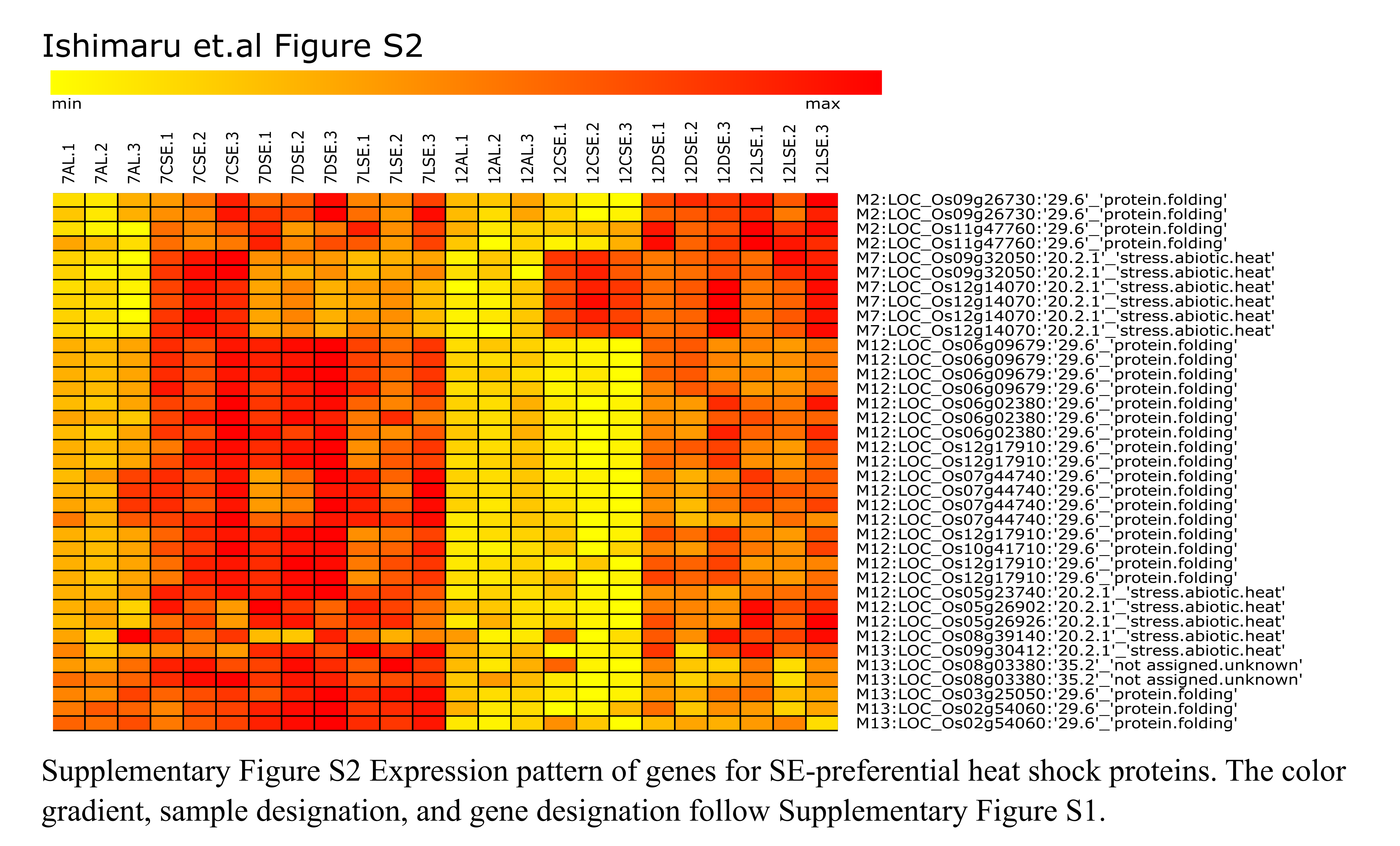

Supplement: Supplementary file 2 — Supplementary file2 (TIF 1931 kb) [file 11103_2021_1225_MOESM2_ESM.tif]

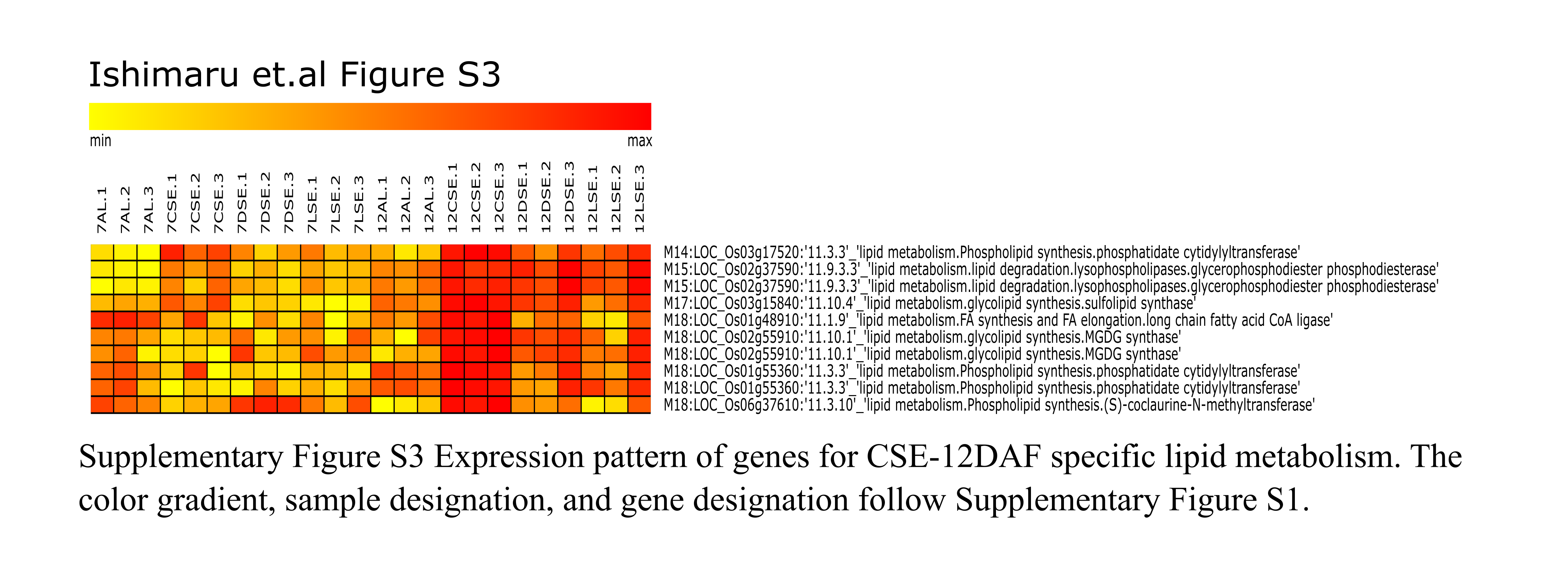

Supplement: Supplementary file 3 — Supplementary file3 (TIF 1128 kb) [file 11103_2021_1225_MOESM3_ESM.tif]

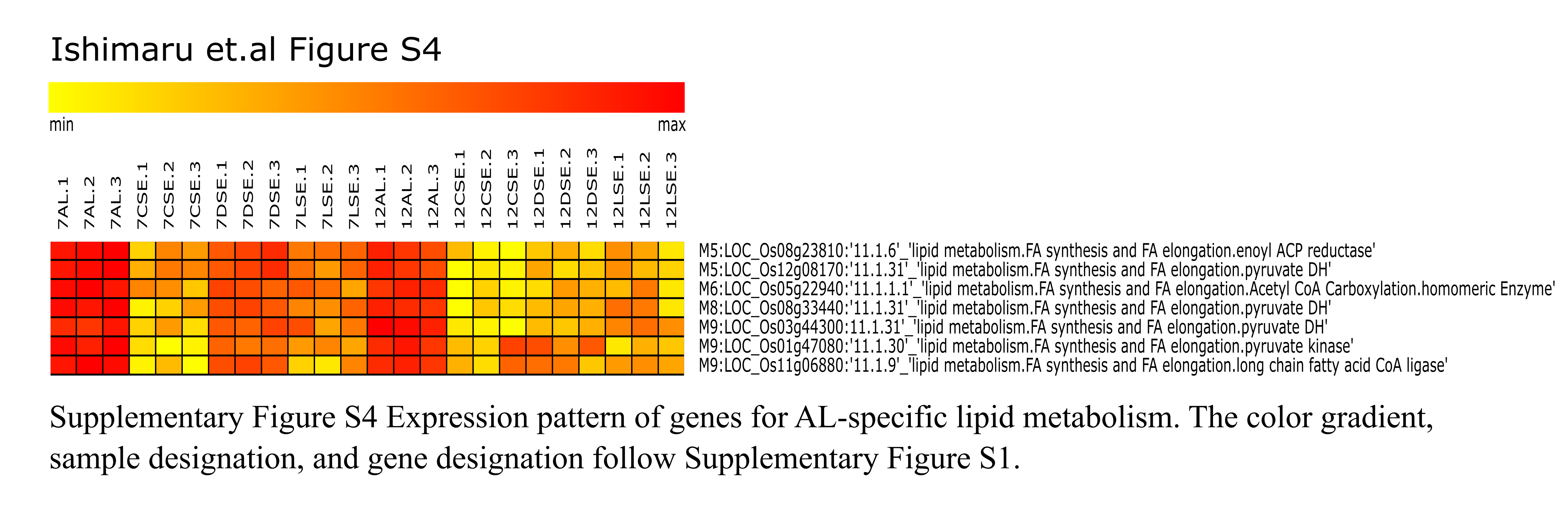

Supplement: Supplementary file 4 — Supplementary file4 (TIF 1006 kb) [file 11103_2021_1225_MOESM4_ESM.tif]

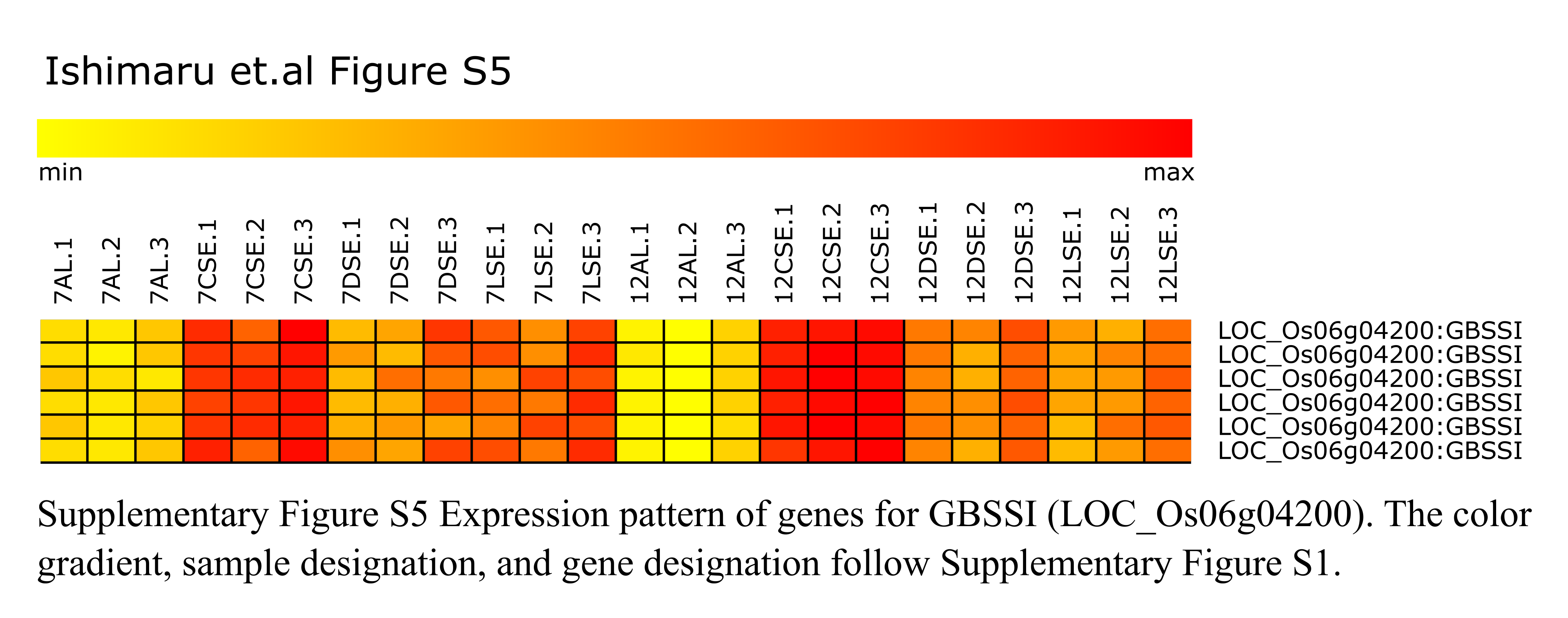

Supplement: Supplementary file 5 — Supplementary file5 (TIF 662 kb) [file 11103_2021_1225_MOESM5_ESM.tif]

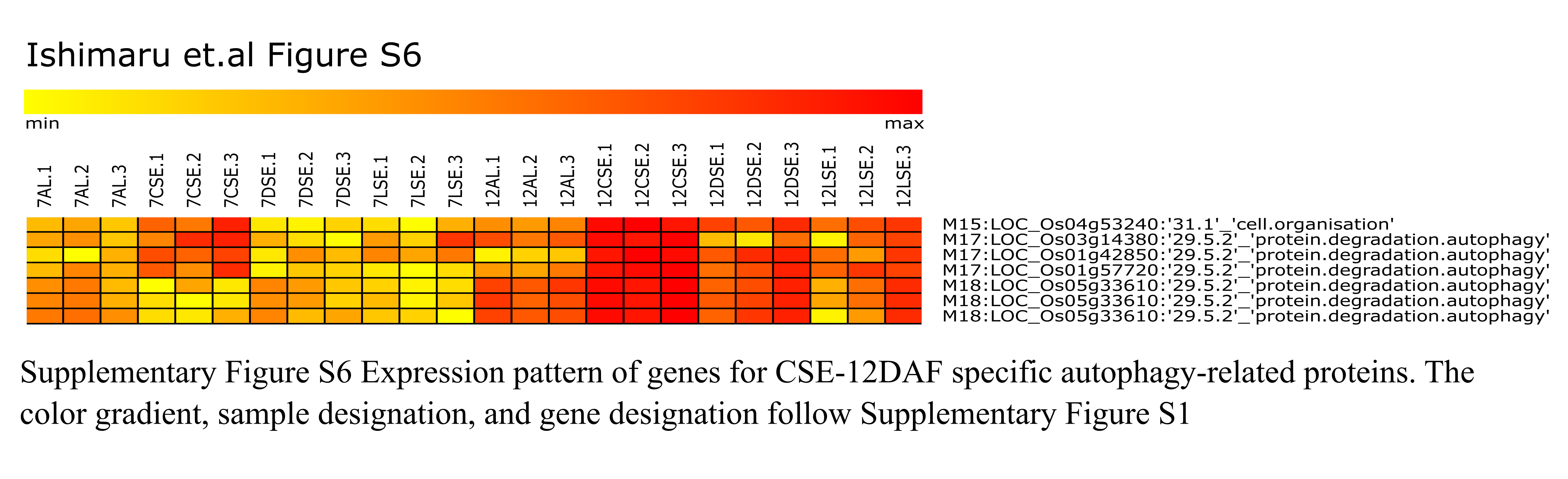

Supplement: Supplementary file 6 — Supplementary file6 (TIF 772 kb) [file 11103_2021_1225_MOESM6_ESM.tif]

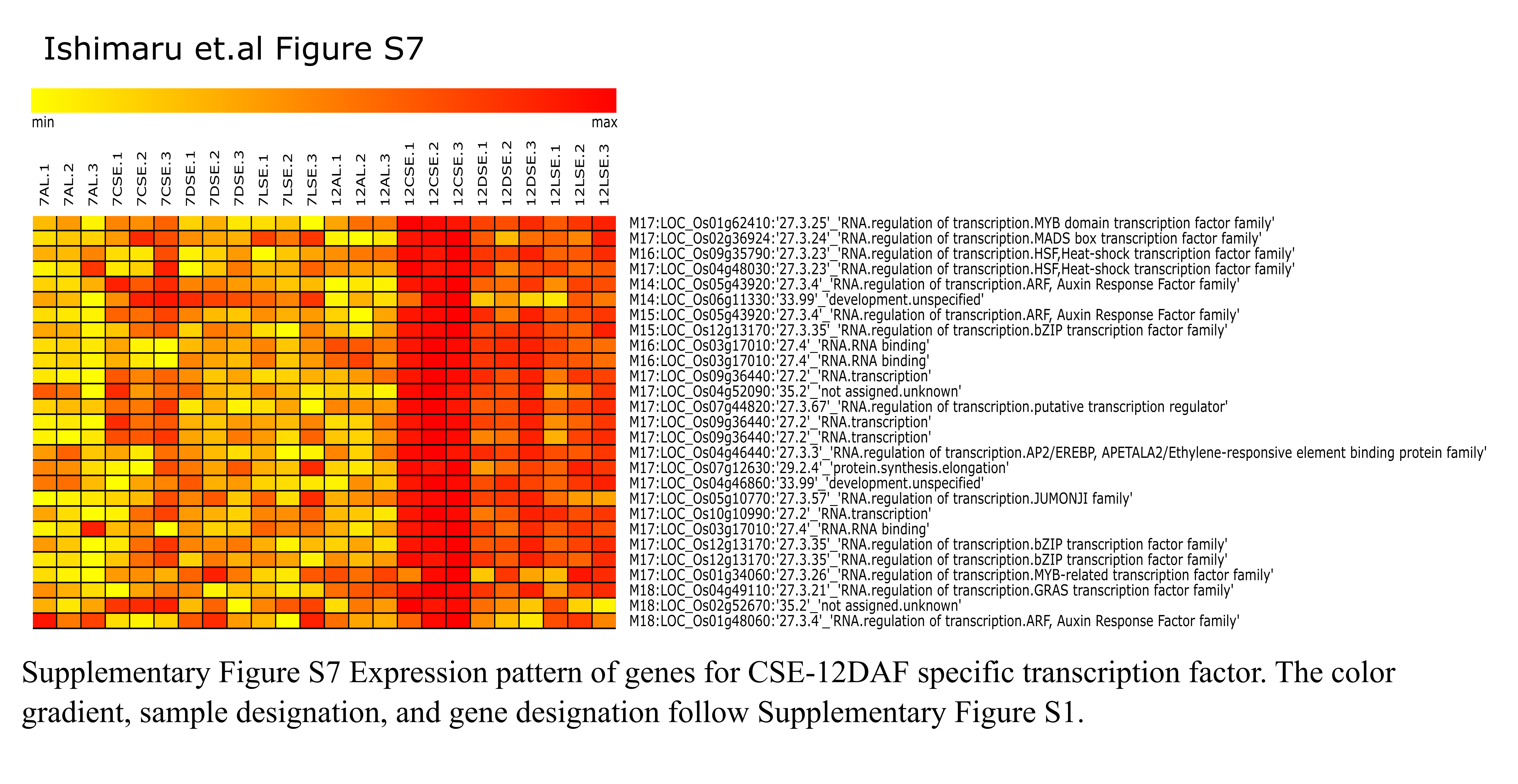

Supplement: Supplementary file 7 — Supplementary file7 (TIF 1983 kb) [file 11103_2021_1225_MOESM7_ESM.tif]

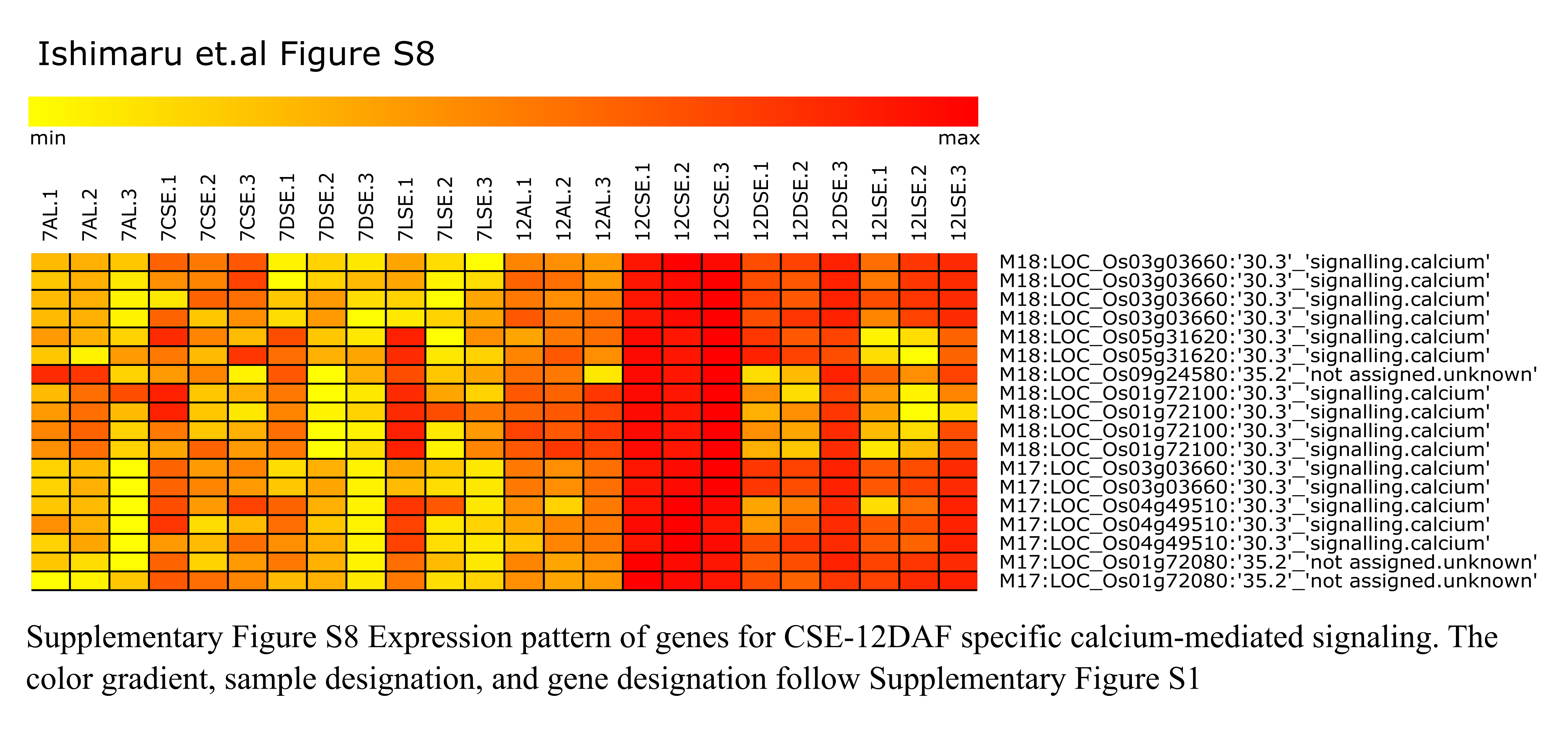

Supplement: Supplementary file 8 — Supplementary file8 (TIF 1395 kb) [file 11103_2021_1225_MOESM8_ESM.tif]

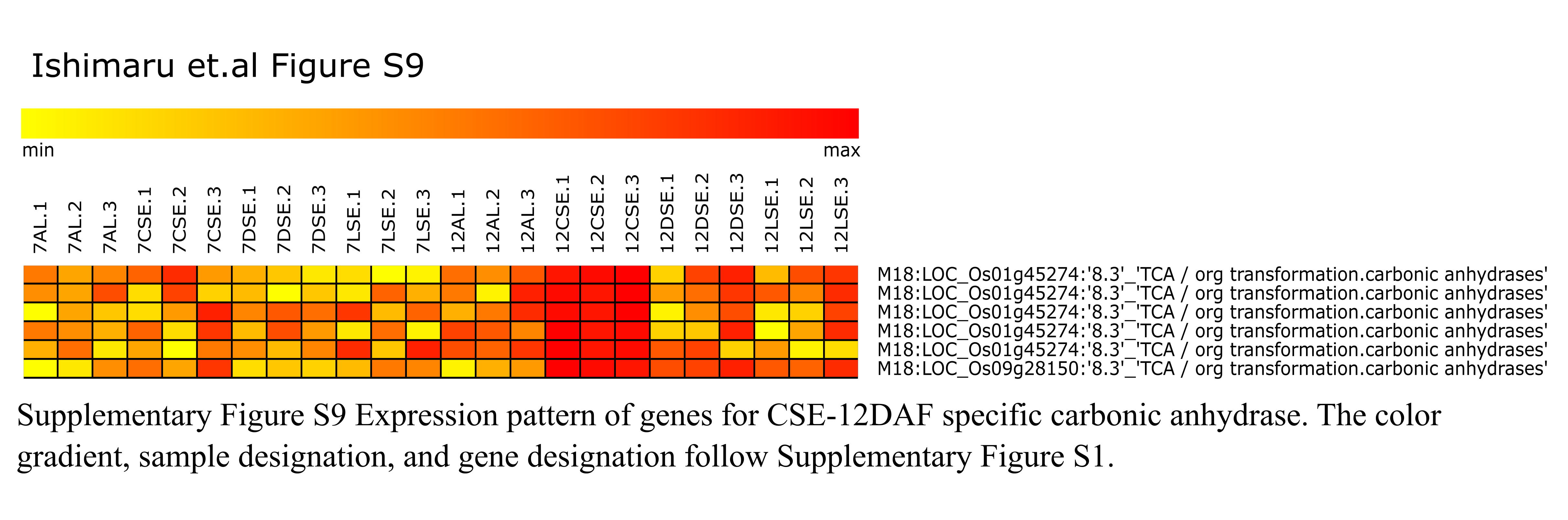

Supplement: Supplementary file 9 — Supplementary file9 (TIF 843 kb) [file 11103_2021_1225_MOESM9_ESM.tif]

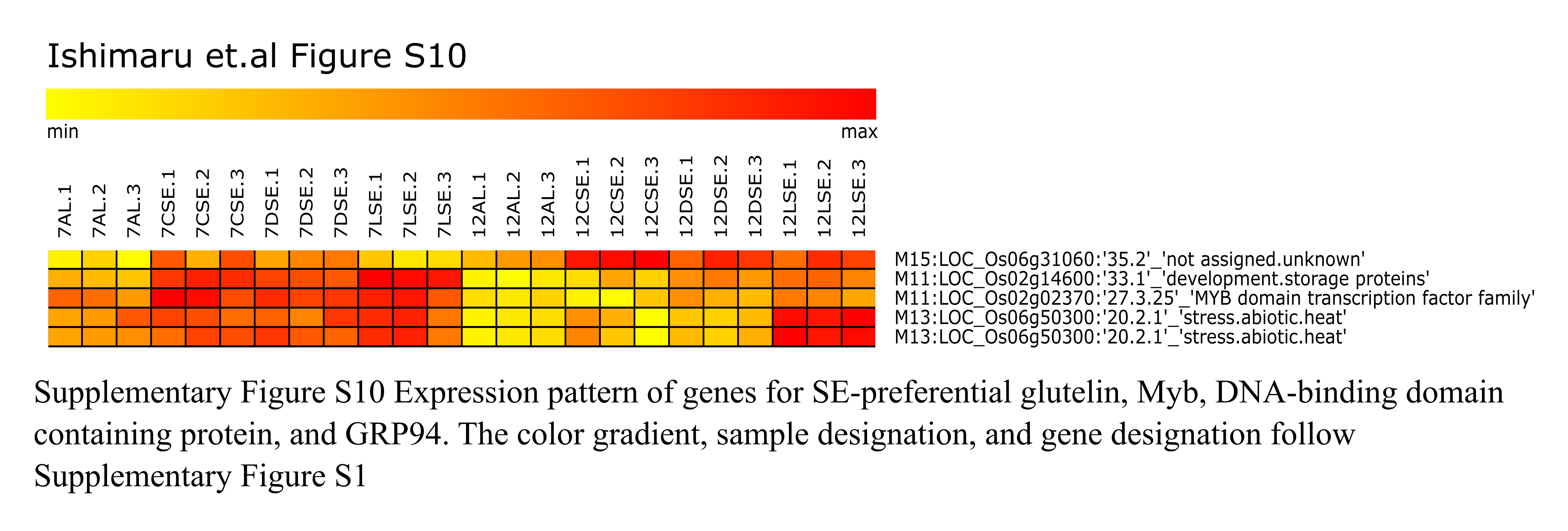

Supplement: Supplementary file 10 — Supplementary file10 (TIF 783 kb) [file 11103_2021_1225_MOESM10_ESM.tif]

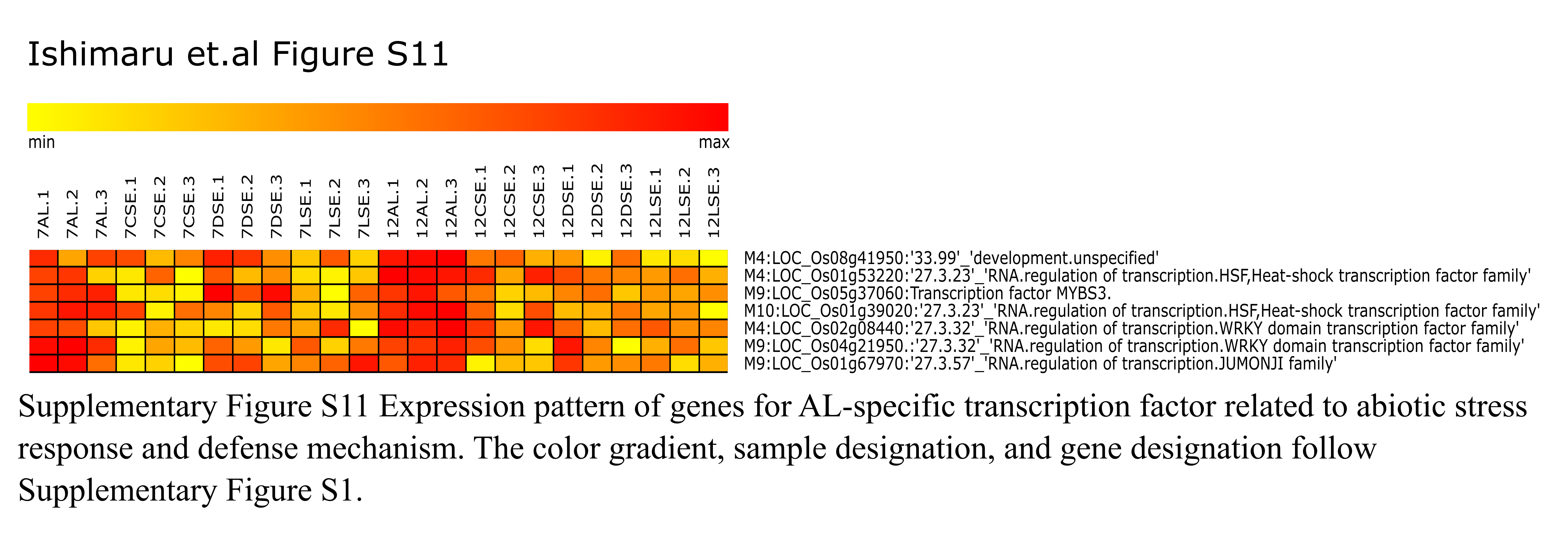

Supplement: Supplementary file 11 — Supplementary file11 (TIF 955 kb) [file 11103_2021_1225_MOESM11_ESM.tif]

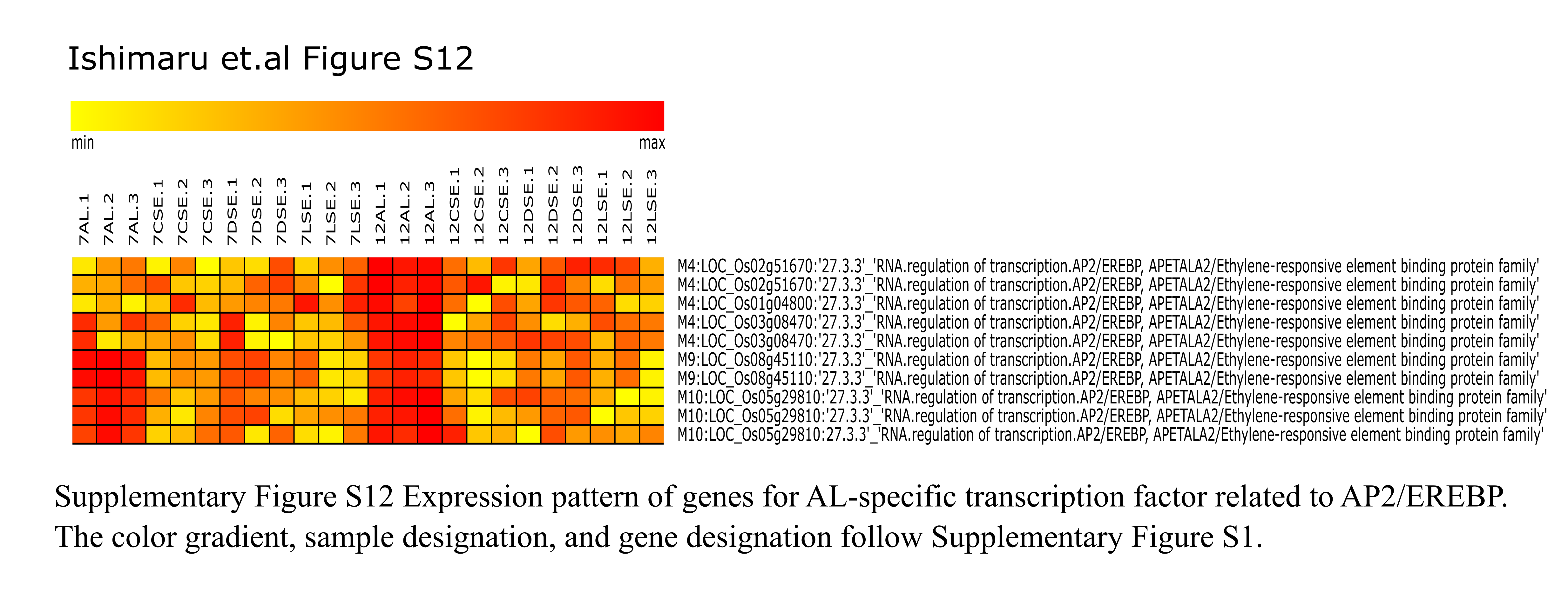

Supplement: Supplementary file 12 — Supplementary file12 (TIF 1461 kb) [file 11103_2021_1225_MOESM12_ESM.tif]

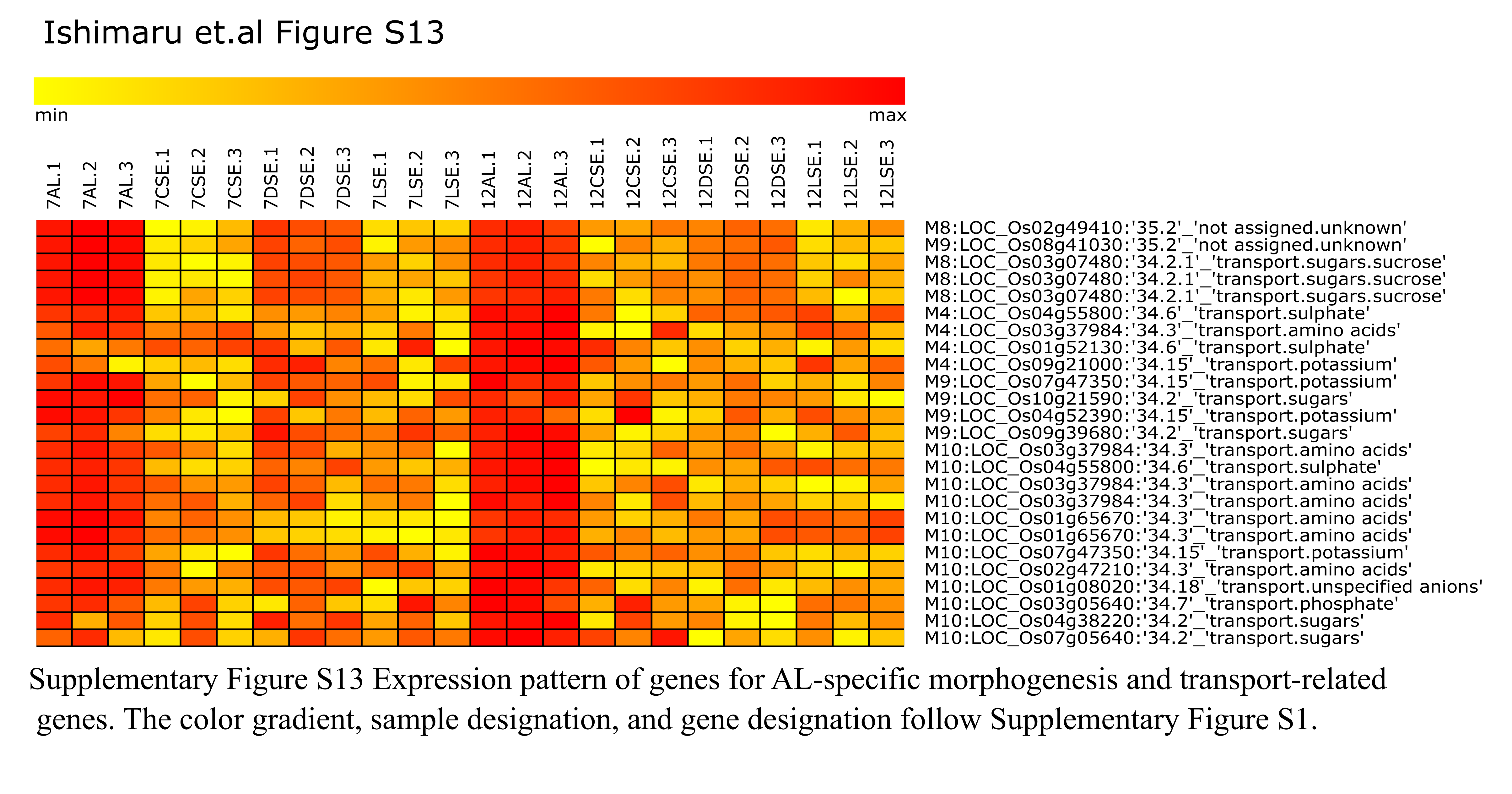

Supplement: Supplementary file 13 — Supplementary file13 (TIF 1721 kb) [file 11103_2021_1225_MOESM13_ESM.tif]
